# Evolution of a Restriction Factor by Domestication of a Yeast Retrotransposon

**DOI:** 10.1093/molbev/msae050

**Published:** 2024-03-05

**Authors:** J Adam Hannon-Hatfield, Jingxuan Chen, Casey M Bergman, David J Garfinkel

**Affiliations:** Department of Biochemistry and Molecular Biology, University of Georgia, Athens, GA, USA; Institute of Bioinformatics, University of Georgia, Athens, GA, USA; Institute of Bioinformatics, University of Georgia, Athens, GA, USA; Department of Genetics, University of Georgia, Athens, GA, USA; Department of Biochemistry and Molecular Biology, University of Georgia, Athens, GA, USA

**Keywords:** retrotransposon, host defense, *Saccharomyces*, domesticated gene

## Abstract

Transposable elements drive genome evolution in all branches of life. Transposable element insertions are often deleterious to their hosts and necessitate evolution of control mechanisms to limit their spread. The long terminal repeat retrotransposon Ty1 prime (Ty1′), a subfamily of the Ty1 family, is present in many *Saccharomyces cerevisiae* strains, but little is known about what controls its copy number. Here, we provide evidence that a novel gene from an exapted Ty1′ sequence, domesticated restriction of Ty1′ relic 2 (*DRT2*), encodes a restriction factor that inhibits Ty1′ movement. *DRT2* arose through domestication of a Ty1′ *GAG* gene and contains the C-terminal domain of capsid, which in the related Ty1 canonical subfamily functions as a self-encoded restriction factor. Bioinformatic analysis reveals the widespread nature of *DRT2*, its evolutionary history, and pronounced structural variation at the Ty1′ relic 2 locus. Ty1′ retromobility analyses demonstrate *DRT2* restriction factor functionality, and northern blot and RNA-seq analysis indicate that *DRT2* is transcribed in multiple strains. Velocity cosedimentation profiles indicate an association between Drt2 and Ty1′ virus-like particles or assembly complexes. Chimeric Ty1′ elements containing *DRT2* retain retromobility, suggesting an ancestral role of productive Gag C-terminal domain of capsid functionality is present in the sequence. Unlike Ty1 canonical, Ty1′ retromobility increases with copy number, suggesting that C-terminal domain of capsid–based restriction is not limited to the Ty1 canonical subfamily self-encoded restriction factor and drove the endogenization of *DRT2*. The discovery of an exapted Ty1′ restriction factor provides insight into the evolution of the Ty1 family, evolutionary hot-spots, and host–transposable element interactions.

## Introduction

Long terminal repeat (LTR) retrotransposons comprise a major class of retrovirus-like transposable elements (TEs) that transpose via an RNA intermediate. Seven LTR retrotransposon families (Ty1, Ty2, Ty3, Ty4, Ty5, Ty3_1p, and Tsu4) have been identified in the model eukaryotic microbe *Saccharomyces cerevisiae* (Kim et al. 1998; Carr et al. 2012; Bergman 2018; O'Donnell et al. 2023). The Ty1 family is the most intensively studied and includes the closely related Ty1 prime (Ty1′) and Ty1 canonical (Ty1c) subfamilies. A reconstruction of ancestral Ty1′ and Ty1c Gag and Pol proteins show 79% and >90% identity, respectively (Kim et al. 1998; Czaja et al. 2020). While much of our mechanistic understanding of retrotransposon biology comes from Ty1c (Curcio et al. 2015), Ty1′ has the hallmarks of a competent retrotransposon. Ty1c is transcribed from 5′ LTR to 3′ LTR and contains 2 open reading frames (ORFs): *GAG* and *POL*. Synthesis of Pol occurs via a programed +1 frame shift in the *GAG* coding sequence (Clare et al. 1988). The 2 primary protein products, p49 Gag and p199 Gag-Pol, are proteolytically processed to mature Gag, protease (PR), integrase (IN), and reverse transcriptase (RT). Ty1c Gag contains retroviral capsid N-terminal domain (CA-NTD) and C-terminal domain (CA-CTD) that are required for VLP assembly and retrotransposition (Tucker et al. 2015; Cottee et al. 2021). Gag also contains a distinct nucleic acid chaperone region C-terminal to the capsid required for Ty1c RNA interactions such as dimerization and packaging (Nishida et al. 2015; Gumna et al. 2019; Gumna et al. 2021). Reverse transcription occurs in mature VLPs and requires the concerted activity of RT and IN, with Ty1c RNA serving as the template (Curcio et al. 2015). A preintegration complex minimally containing Ty1c cDNA and IN is imported into the nucleus with the aid of a nuclear localization signal in IN (Kenna et al. 1998; Moore et al. 1998). Integration occurs preferentially near genes transcribed by RNA polymerase III due to an interaction between IN and a subunit of RNA Pol III (Bridier-Nahmias et al. 2015; Cheung et al. 2016).

Eukaryotic genomes are constantly coevolving with their TEs. Hosts have evolved multiple mechanisms to protect against the potentially deleterious effects of transposition events, including DNA methylation, repressive chromatin modifications, RNAi, and APOBEC mRNA editing (Friedli and Trono 2015; Goodier 2016). Although *S. cerevisiae* lacks these defense pathways, Ty1c transposition is restrained by a unique and effective TE control mechanism, referred to as copy number control (CNC). CNC is the phenomenon wherein Ty1c mobility decreases as the total number of Ty1c copies in the genome increases (Garfinkel et al. 2003). CNC is mediated by the Ty1c protein p22, which arises from an internal transcript initiation site (Ty1i) in Gag and contains the CA-CTD but lacks the CA-NTD (Saha et al. 2015). p22 interacts with full-length Gag to disrupt the capsid function of VLP assembly and maturation (Nishida et al. 2015; Saha et al. 2015; Cottee et al. 2021). Thus, p22 displays properties of a *trans-*dominant restriction factor of Ty1c retrotransposition. Whether Ty1′ encodes a similar CNC mechanism or if there are other novel control mechanisms that act on Ty1′ remains to be determined.

Advances in genomic studies of *S. cerevisiae* have revealed many insights into the dynamics of host and TE interactions by capturing the TE content variation at the species level (Liti et al. 2009; Carr et al. 2012; Bleykasten-Grosshans et al. 2013, 2021; Peter et al. 2018; O'Donnell et al. 2023). LTRs within full-length Ty elements frequently undergo recombination creating solo LTRs, which are abundant in the genome and often found at high allele frequency in *S. cerevisiae* populations (Kim et al. 1998; Carr et al. 2012). In contrast, full-length and truncated Ty elements with internal coding sequences are relatively rare in *S. cerevisiae* genomes and are typically found at low allele frequencies (Carr et al. 2012; O'Donnell et al. 2023). Two truncated Ty1′ elements on chromosome IV (called relics 1 and 2) containing *GAG* sequences were previously identified as being at unusually high allele frequency in draft genomes of multiple *S. cerevisiae* strains (Bleykasten-Grosshans et al. 2013). PacBio long-read sequencing subsequently revealed a conserved structure for both relics in diverse *S. cerevisiae* strains (Czaja et al. 2020). Intriguingly, the relic 2 locus contains a nearly complete Ty1′ *GAG* sequence including the CA-CTD, which has the potential to act as a repressor similar to how it functions in Ty1c retrotransposition. Here, we investigate the history and function of the relic 2 locus and show that it encodes a novel gene, *DRT2*, which is derived from a domesticated Ty1′ *GAG* and exhibits properties of a restriction factor similar to Ty1c p22.

## Results

### Structure of the *S. cerevisiae* Relic 2 Locus and *DRT2* Gene

**Fig. 1.**
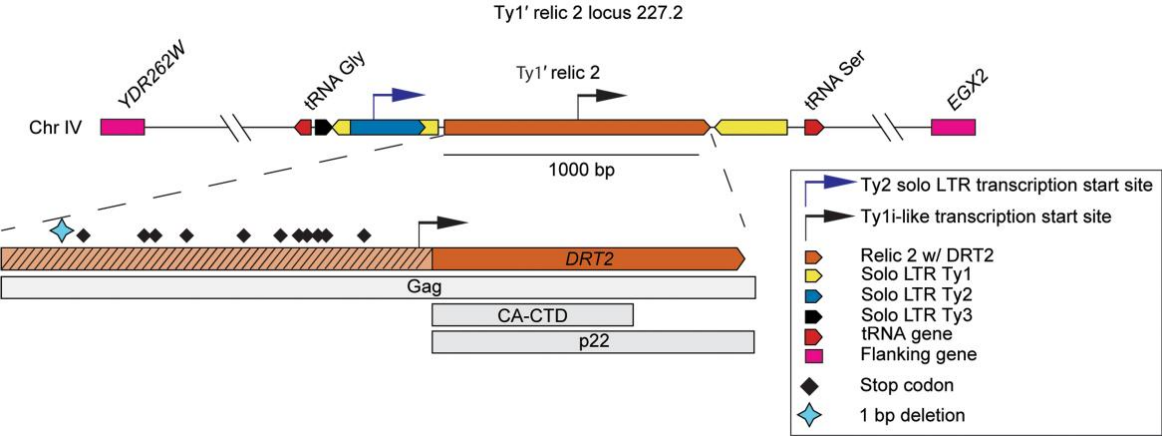
Organization of the relic 2 locus on chromosome IV in UWOPS05-227.2. The relic 2 Ty1′ insertion is flanked upstream by Ty1, Ty2, and Ty3 solo LTR sequences, tRNA Gly (YNCD0020W), and the coding gene *YDR262W* and flanked downstream by Ty1 solo LTR sequence, tRNA Ser (YNCD0019C), and the coding gene *EXG2*. The approximate locations of Ty2 solo LTR and Ty1i-like transcription start sites are labeled with blue and black arrows, respectively. Within the relic 2 Ty1′ sequence, a 1 bp deletion relative to Ty1′ *GAG* (blue diamond) causes in-frame stop codons (black diamonds) upstream of *DRT2*. Regions homologous to Ty1c *GAG*, the CA-CTD coding region, and the p22 protein are shown as gray boxes.

Relic 2 resides at a fixed chromosomal position in multiple strains (Bleykasten-Grosshans et al. 2013; Czaja et al. 2020). However, relic 2 fine structure, variation, and potential functions remain unexplored. We define the relic 2 locus as the region on chromosome IV bracketed by tRNA Gly (YNCD0020W) upstream and tRNA Ser (YNCD0019C) downstream (Fig. 1). The Ty1′ insertion at the relic 2 locus is absent in the S288C reference strain but is present in many additional strains including the wild Malaysian strain UWOPS05-227.2 (227.2) (Czaja et al. 2020). We chose 227.2 as our reference strain in the current study because it has a relatively simple relic 2 structure and lacks any other complete Ty elements in its genome, which facilitated functional analysis. Fine structure analysis of the 227.2 relic 2 locus revealed a 1,187 bp segment derived from Ty1′ *GAG* bordered by additional Ty1, Ty2, and Ty3 solo LTR fragments and flanking tRNA genes (Fig. 1). A 1 bp deletion caused multiple in-frame stop codons in the 5′ region of *GAG* but left an intact ORF in *GAG* spanning the CA-CTD region. *DRT2* is defined as the gene encoding this intact ORF based on the functional evidence presented in this study. The *DRT2* ORF in 227.2 spans a region homologous to nearly the entire p22 region in Ty1c, including the CA-CTD region that has been shown to encode restriction factor activity in Ty1c (supplementary fig. S1, Supplementary Material online) (Tucker et al. 2015; Cottee et al. 2021).

### Evolution of the *S. cerevisiae* Relic 2 Locus

To better understand the history and diversity of the relic 2 locus, we developed a novel approach to locally assemble this region from long-read whole genome sequencing data sets (see Materials and Methods and supplementary fig. S2A, Supplementary Material, online for details) and applied it to 100 strains of *S. cerevisiae* (Istace et al. 2017; Yue et al. 2017; Czaja et al. 2020; Linder et al. 2020; Basile et al. 2021; Bendixsen et al. 2021; Lee et al. 2022), including 6 new PacBio data sets from diverse Chinese lineages (supplementary table S1, Supplementary Material online). Briefly, we extracted flanking or spanning reads that mapped with high quality to the *S. cerevisiae* 227.2 relic 2 locus and used them as input for de novo assembly with 2 assemblers: Flye (Kolmogorov et al. 2019) and wtdbg2 (Ruan and Li 2020). By using long reads mapped to regions flanking the relic 2 locus, this approach allows assembly of haplotypes that either contain or lack the relic 2 Ty1′ insertion. Consistency of both assemblies, alignment of unassembled raw reads to local assemblies, and alignment of local assemblies to the relic 2 locus from the whole genome assembly of reference strain 227.2 were used to evaluate the quality of local assemblies. Seventy-nine strains passed our quality control analysis and were regarded as having high-quality relic 2 locus assemblies. We selected the default polished Flye assemblies for these 79 strains for further sequence analysis including annotation of Ty elements and tRNA genes. One sample, strain ADQ, failed tRNA annotation with 1 missing flanking tRNA and was excluded from further analysis. In addition, 5 strains have been sequenced in multiple publications and duplicate strains were removed from final analysis. In total, 73 nonredundant samples with high-quality local assemblies were used to investigate sequence and structural variation in the relic 2 locus.

**Fig. 2.**
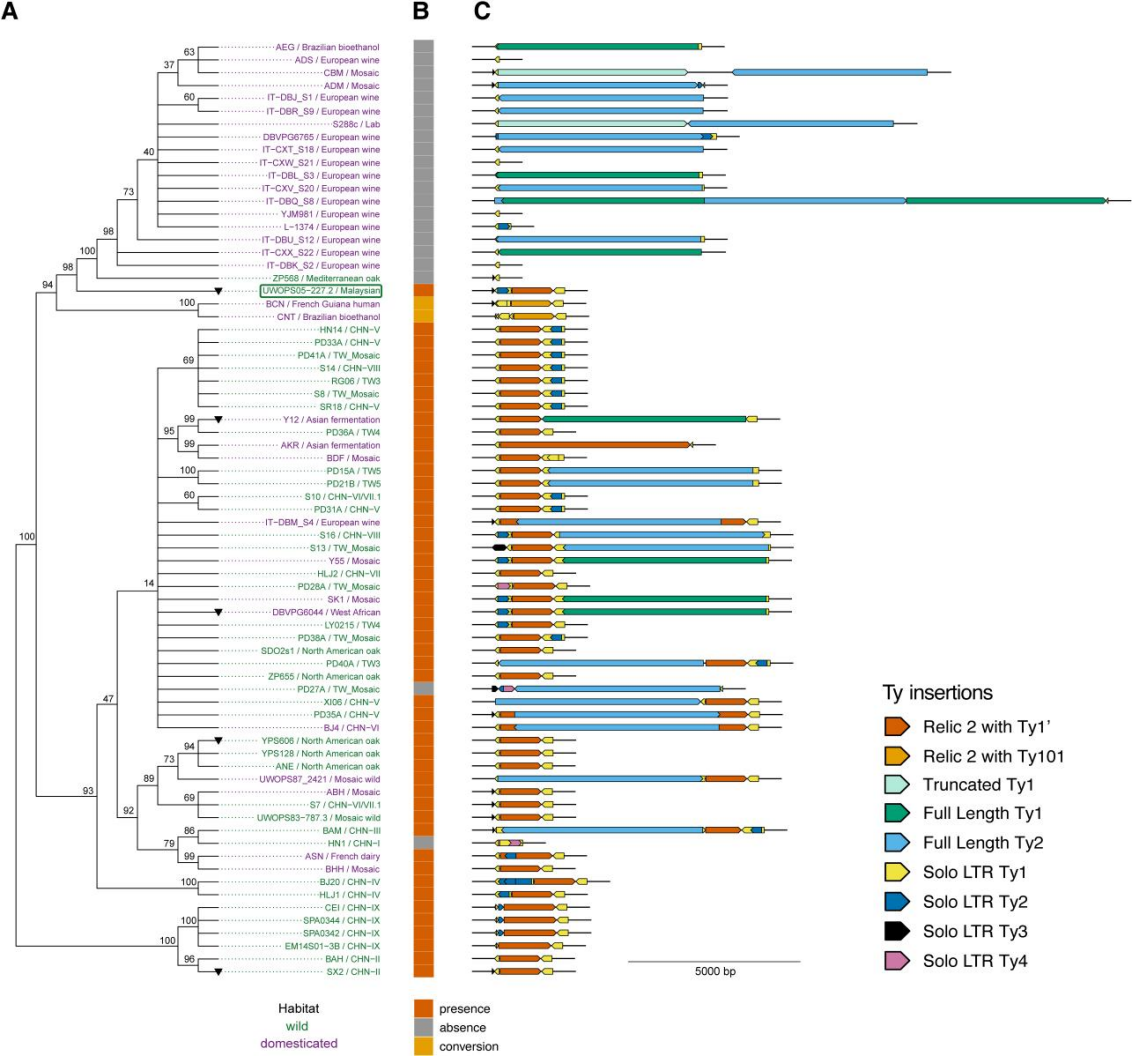
Evolution of the relic 2 locus. A) ML phylogenetic tree based on the concatenated sequence 1 kb upstream and 1 kb downstream of the tRNA genes from local assemblies of the relic 2 locus. The phylogeny was rooted with the clade containing the most ancient *S. cerevisiae* lineage (China IX/Taiwan). Branch lengths do not represent evolutionary distance, and branches <1e^−05^ were omitted to simplify the visualization. Taxa in green indicate strains from wild isolations; taxa in purple represent strains from domesticated/human associated lineages. Numbers at internal nodes represent bootstrap support values based on 100 replicates. The 227.2 reference is highlighted in the box. Five strains used for functional analysis of *DRT2* alleles are indicated with black triangles. B) Color stripe showing the presence (orange), absence (gray), or gene conversion (gold) of the relic 2 Ty1′ insertion in each strain. BCN and CNT contain a truncated insertion structurally like relic 2 Ty1′, but with a sequence haplotype from the Ty101 subfamily, and represent putative gene conversion events. C) Ty content of relic 2 locus. Arrowheads indicate the orientation of annotated Ty fragments.

Using these 73 high-quality local assemblies, we reconstructed the evolutionary history of the genomic region containing the relic 2 locus using a maximum likelihood (ML) phylogenetic approach applied to the concatenated 1 kb of sequence upstream or downstream of the flanking tRNA genes (Fig. 2A). This approach allowed us to study the evolution of the relic 2 locus independent of the presence/absence of the relic 2 Ty1′ insertion or other sequence variation in the relic 2 locus. The resulting phylogeny was rooted using a clade that contained strains from the China IX/Taiwanese lineage, which has been shown to be the deepest ancestral lineage of *S. cerevisiae* (Duan et al. 2018; Peter et al. 2018). Next, we determined the presence or absence of the relic 2 Ty1′ insertion in each strain based on whether it contained a truncated Ty1′ fragment in the forward orientation (Fig. 2B). To differentiate bona fide relic 2 Ty1′ insertions from other truncated insertions in the Ty1 family, we reconstructed a phylogenetic tree of all truncated elements from the Ty1 family found in the relic 2 locus (supplementary fig. S2B, Supplementary Material online). Among the 73 strains with high-quality local assemblies, we found that 50 strains clearly contained the relic 2 Ty1′ insertion containing *DRT2*, while 23 lacked the relic 2 Ty1′ insertion. Two strains lacking the relic 2 Ty1′ insertion (S288C and CBM) share a truncated Ty1c insertion. Two additional strains that lack the relic 2 Ty1′ insertion (BCN and CNT) contained a truncated insertion structurally like relic 2 Ty1′, but with a sequence haplotype from the Ty101 subfamily (Bleykasten-Grosshans et al. 2021). We then visualized the Ty content between the tRNA genes on the tree, which revealed substantial structural variation in the relic 2 locus including many Ty insertions in addition to the truncated Ty1′ insertion (Fig. 2C). High Ty activity at the relic 2 locus is expected, as sequences adjacent to tRNA genes are preferred targets of Ty1, Ty2, Ty3, and Ty4 insertions (Bridier-Nahmias et al. 2015; Patterson et al. 2019).

Strains containing relic 2 were spread widely across divergent lineages, including most Chinese lineages, French dairy, Asian fermentation, West African, North American oak, and several mosaic lineages. The presence of relic 2 in strains from the most ancient Asian lineages of *S. cerevisiae* (EM14S01-3B, CEI, BAH and SX2) and in all wild lineages (except European oak and CHN-I) suggests that relic 2 likely existed in the common ancestor of all global *S. cerevisiae* lineages. We infer 3 losses of relic 2 due to deletion events: one on the lineage leading to a clade containing European wine, European oak, and the lab strain S288c, one on the lineage leading to the mosaic strain PD27A, and one on the lineage leading to the CHN-I strain HN1. The 2 S. American strains with truncated Ty101 sequences (BCN and CNT) phylogenetically cluster with relic 2 Ty1′ containing strains. The phylogenetic clustering and similarity in structure with bona fide relic 2 Ty1′ containing strains raises the possibility that the ancestor of BCN and CNT had a relic 2 Ty1′ that subsequently underwent gene conversion with an element from the Ty101 subfamily (Roeder and Fink 1982), which is found only in S. America (Bleykasten-Grosshans et al. 2021). This putative conversion event can be classified as a 4th loss event, as it eliminates the Ty1′ haplotype that defines bona fide relic 2 containing haplotypes.

Although the structure and content of Ty elements in relic 2 Ty1′ positive strains varied across lineages, the most common structure of the relic 2 locus contained a truncated Ty1′ containing *GAG* sequence in the forward orientation, a 100 bp partial Ty1 solo LTR upstream, and a complete Ty1 solo LTR downstream in reverse orientation. This relic 2 structure was shared by 11 strains (BAH, SX2, UWOPS83-787.3, ABH, YPS128, YPS606, ANE, ZP655, HLJ2, SDO2s1, and BHH). Other strains contain Ty insertions either upstream of the *GAG* sequences (227.2, HLJ1, BJ20, and UWOPS87_2421) or downstream (BDF, Y12, and HN14), or both (BAM, DBVPG6044, SK1, and Y55). Three strains contained *GAG* segments that are interrupted by Ty insertions, but importantly all occur outside the *DRT2* ORF (IT-DBM_S4, ASN, and BJ4). In addition, elongated but not full-length Ty1′ insertions were detected in 5 strains (SPA0344, SPA0342, CEI, EM14S01-3B, and AKR), raising the possibility that these represent more ancestral states of relic 2. The relic 2 loci in strains SPA0344, SPA0342, CEI, and EM14S01-3B included an additional LTR upstream of the *GAG* segment, and AKR relic 2 is comprised of a *GAG* segment containing several stop codons upstream of *DRT2*, a *POL* segment, and a downstream LTR. These observations, in conjunction with the distinct CHN-IX/Taiwanese group in the phylogeny of relic 2 (supplementary fig. S2B, Supplementary Material online), suggest that SPA0344, SPA0342, CEI, and EM14S01-3B display an ancestral state of relic 2.

Of the 50 strains that contain relic 2, 40 were assembled from PacBio or recent Oxford Nanopore Technologies (ONT) long reads and had high-quality primary sequence data of the *DRT2* ORF. Strikingly, the *DRT2* ORF remains intact in all 40 strains, with a 1 bp deletion at the terminus of the *DRT2* region in strain 227.2 that extends the coding region by 2 amino acids. Codon-based ML analysis (Yang 2007) of the pattern of point substitution in the *DRT2* ORF upstream of the 2 additional amino acid residues specific to strain 227.2 suggests it is evolving as a coding region under purifying selection (dN/dS = 0.83968). Together, our evolutionary analysis demonstrates that relic 2 is an ancient feature of the *S. cerevisiae* genome and the relic 2 locus has undergone extensive structural variation including secondary loss and additional Ty insertion events in some strains. Nevertheless, a conserved *DRT2* ORF is found in all strains that maintain relic 2, supporting the hypothesis that the *DRT2* ORF is a functional coding region exapted from an ancient Ty1′ insertion event.

### *DRT2* Restricts Ty1′ Retromobility

**Fig. 3.**
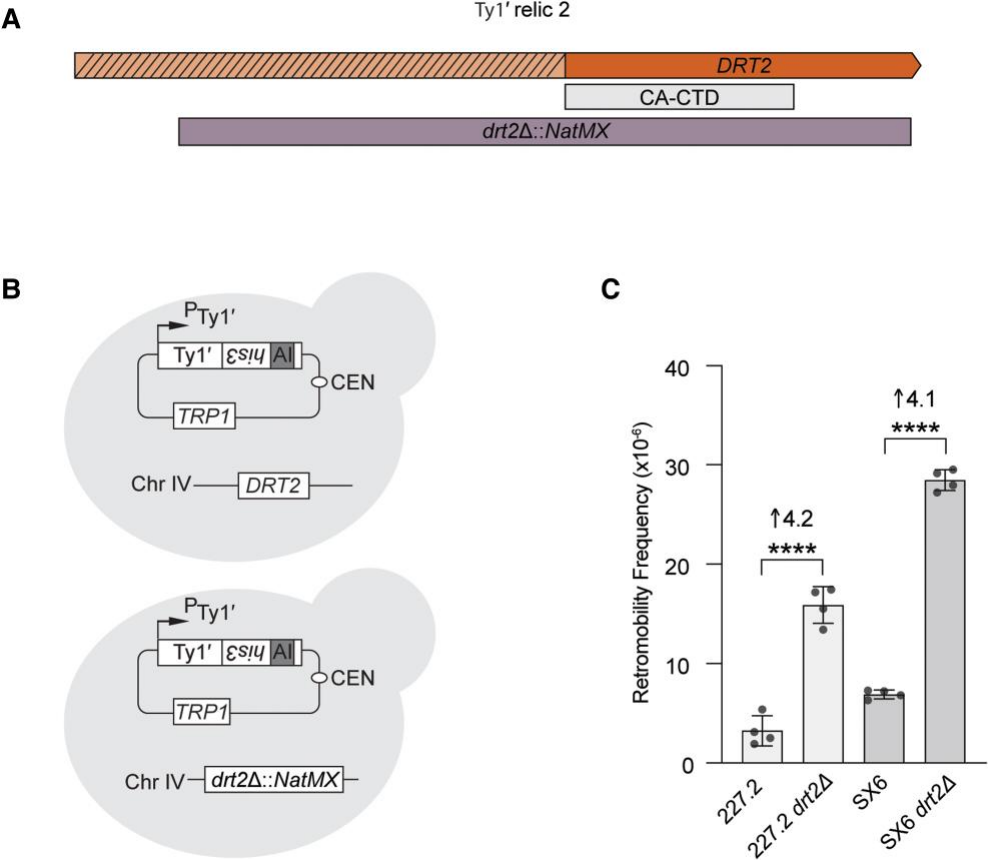
*DRT2* restriction of Ty1′. A) The relic 2 locus with the *DRT2* deletion interval (*drt2Δ*::*NatMX*, purple box) and CA-CTD is indicated. B) Scheme for determining *DRT2* restriction of Ty1′. A Ty1′ reporter element under its endogenous promoter (PTy1′) containing the *his3-AI* retromobility reporter is presented. Ty1′*his3-AI* mobility restores histidine prototrophy in a strain lacking chromosomal *HIS3*. An arrow represents transcription start site. PTy1′ contains a low-copy centromere replication sequence (CEN) and the *TRP1* gene. The 2 strains represent the WT parent containing *DRT2* or the knockout containing *drt2::ΔNatMX.* C) Ty1′*his3-AI* retromobility in isogenic 227.2 or SX6 WT and *drt2Δ* strains. Bars represent the mean of at least 4 independent measurements (circles). Standard deviation is represented by error bars. Fold change compared with WT is shown above bars. Statistical significance measured with Welch's *t*-test comparison of WT with *drt2Δ* strains. ****P < 0.0001 with exact values reported in supplementary table S4, Supplementary Material online.

Based on conservation of *DRT2* in diverse *S. cerevisiae* strains, homology to the Ty1′ Gag CA-CTD (supplementary fig. S1, Supplementary Material online), and known functions of Ty1c CA-CTD as a capsid building block and restriction factor (Nishida et al. 2015; Saha et al. 2015; Tucker et al. 2015; Cottee et al. 2021), we hypothesized that *DRT2* is a domesticated retrotransposon gene that encodes a restriction factor of Ty1′ mobility. To determine if *DRT2* affects Ty1′ retromobility, we first constructed an isogenic 227.2 derivative for functional analyses by deleting *HIS3* and mutating *FLO8* and *TRP1* from the Y3629 parent strain (Cubillos et al. 2009). This strain and its derivatives are used throughout this study for further genetic and biochemical analyses, with full strain list details available in supplementary table S2, Supplementary Material online. *DRT2* was replaced in this strain with the antibiotic resistance cassette *KanMX* in 227.2 (Fig. 3A) (Wach et al. 1994). We constructed a low-copy centromere-based Ty1′*his3-AI* reporter plasmid (pBDG1785) containing a full-length Ty1′ from the reference strain S288C (YBLWTy1-1, https://www.yeastgenome.org/, SGD ID:S000006808) that has been shown to be transcriptionally active (Morillon et al. 2002). The level of Ty1′*his3-AI* retromobility was determined with reporter plasmid Ty1′*his3-AI* in wild-type (WT) and *drt2-Δ::KanMX* strains as described previously for Ty1c (Curcio and Garfinkel 1991). Overall, the frequency of His^+^ colony formation detects Ty1 mobility events from de novo retrotransposition insertion or from a minor homologous recombination pathway where Ty1 cDNA recombines with plasmid borne or genomic Ty1 sequences (Sharon et al. 1994). Additional details on plasmids used in this study are available in supplementary table S3, Supplementary Material online. The Ty1′*his3-AI* reporter plasmid was introduced into strains that were isogenic with one another except at the *DRT2* locus (Fig. 3B). In addition to strain 227.2, *DRT2* was deleted in strain SX6, which also lacks Ty1 elements and is representative of the ancestral CHN-II clade (Wang et al. 2012). Quantitative Ty1′ retromobility assays revealed a 4.2- and 4.1-times higher retromobility frequency in 227.2 and SX6, respectively, in the absence of *DRT2* (Fig. 3C). Importantly, the increase in Ty1′ retromobility in both *drt2Δ* strains demonstrates that *DRT2* acts as a Ty1′ restriction factor in *S. cerevisiae* strains with diverse genetic backgrounds. Additionally, in vivo retromobility of the Ty1′*his3-AI* reporter validates that Ty1′ is a functionally competent retrotransposon.

### *DRT2* Expression

**Fig. 4.**
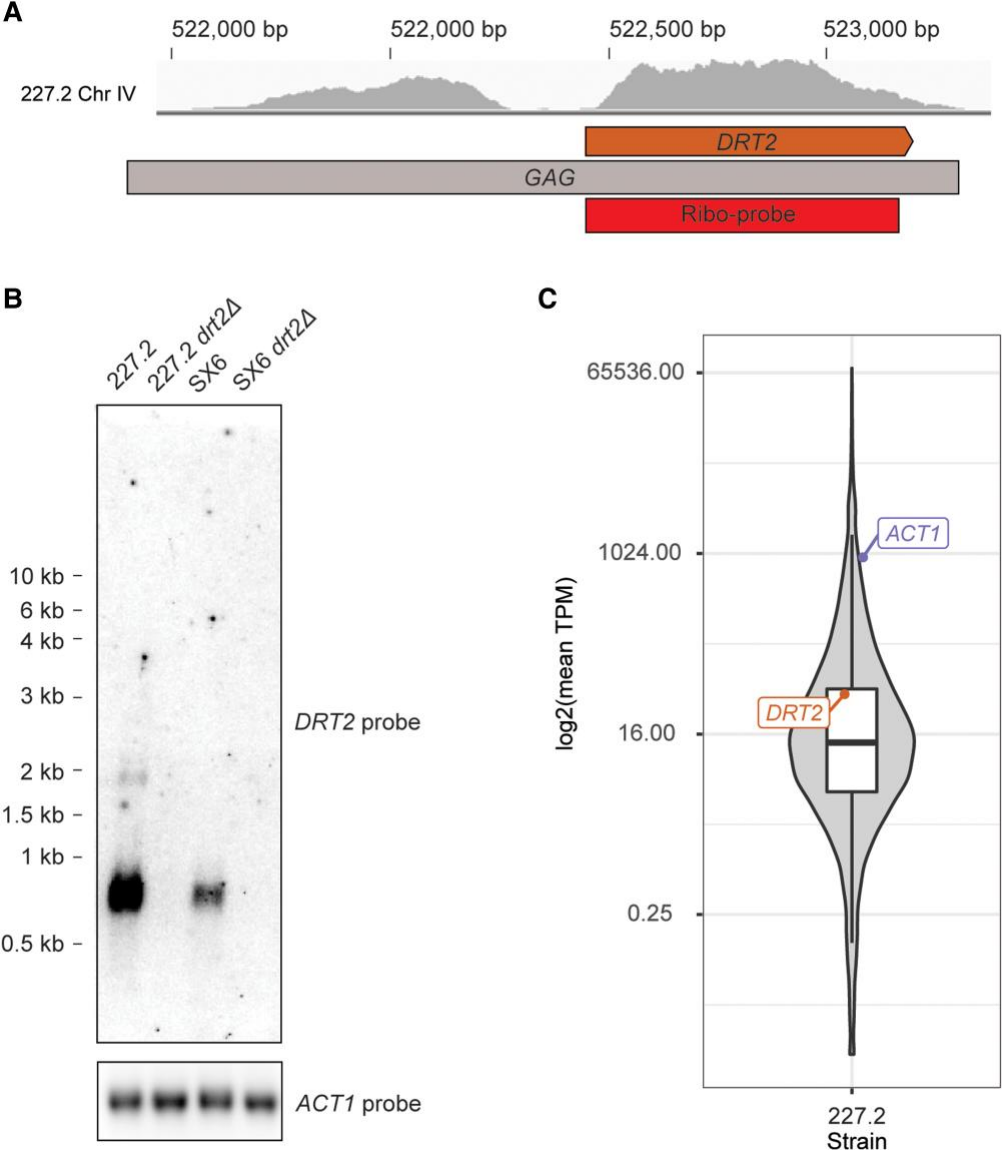
*DRT2* expression. A) Poly(A)^+^ RNA-seq coverage in the relic 2 locus in strain 227.2. Sequences corresponding to *DRT2* orange, *GAG* (gray), and the *DRT2* ribo-probe (red) are noted. B) Northern blot analysis of *DRT2* transcripts. Poly(A)^+^ RNA was isolated from strains 227.2, 227.2 *drt2Δ*, SX6, and SX6 *drt2Δ*, separated by formaldehyde–agarose gel electrophoresis, and hybridized with strand-specific 227.2 *DRT2* or *ACT1*  ^32^P-labeled riboprobes. The band intensities do not correspond to relative abundance of transcripts. RNA size markers are indicated alongside the blot. C) Expression level of *DRT2* relative to other *S. cerevisiae* genes in strain 227.2. Mean TPM for 5,395 *S. cerevisiae* protein-coding genes plus *DRT2* was estimated across 16 runs of poly(A)+ RNA-seq reads in strain 227.2 (Lee et al. 2013) and then log2 transformed and plotted as a boxplot overlayed on a violin plot. The lower and upper edges of the boxplot correspond to the first and third quartiles, whiskers extend to points within ±1.5 the interquartile range, outlying points are not plotted, and *ACT1* and *DRT2* are annotated.

Next, we sought to characterize the *DRT2* transcript and protein product. Based on the sequence upstream of *DRT2* in 227.2, we predicted 2 possible transcription initiation sites. The transcript could initiate in the TATA box contained in the upstream Ty2 solo LTR or from a start site ∼38 bp upstream of the *DRT2* initiator codon (Fig. 1). Precedent for the use of an internal start site comes from the Ty1i transcript in Ty1c, which produces subgenomic transcripts and the self-encoded p22 restriction factor (Liao et al. 1987; Saha et al. 2015; Salinero et al. 2018). These *DRT2* initiation sites are predicted to produce transcripts of ∼2,000 or ∼800 nt, respectively. Both transcripts are predicted to contain the *DRT2* coding sequence. Analysis of poly(A)^+^ RNA-seq data from strain 227.2 (Lee et al. 2013) revealed profiles across *DRT2* that support the presence of distinct transcripts (Fig. 4A). Northern blot analysis revealed a primary *DRT2* transcript ∼800 nt in length in both 227.2 and SX6, suggesting the use of the Ty1i-like internal start site just upstream of *DRT2*. As expected, transcripts were not detected in the *drt2Δ* derivatives. In strain 227.2, a faint ∼2,000 nt transcript was detected that may initiate from the Ty2 solo LTR upstream of *DRT2* (Fig. 4B). To estimate *DRT2* expression level, we quantified transcript abundance in strain 227.2 in units of transcript counts per million (TPM) (Wagner et al. 2012; Lee et al. 2013). This analysis places *DRT2* in the 74th percentile for transcript abundance among 5,395 other protein-coding genes in strain 227.2 (Fig. 4C, supplementary table S5, Supplementary Material online). To analyze endogenous Drt2 protein, we utilized western blotting with a primary antibody raised against the 169 amino acid 227.2 Drt2 sequence (α-Drt2). When Drt2 was overexpressed from the *GAL1* promoter on a multicopy plasmid, a Drt2 protein of the expected size of ∼19.5 kDa was detected. However, endogenous Drt2 was below the limit of detection in whole cell extracts from 227.2 or SX6 under the same western blotting conditions (supplementary fig. S3, Supplementary Material online). Taken together, our results demonstrate that *DRT2* is actively transcribed from a primary transcript ∼800 nt in length in multiple *S. cerevisiae* strains under growth conditions optimal for Ty1c transposition.

### Diverse *DRT2* Alleles Restrict Ty1′ Retromobility

**Fig. 5.**
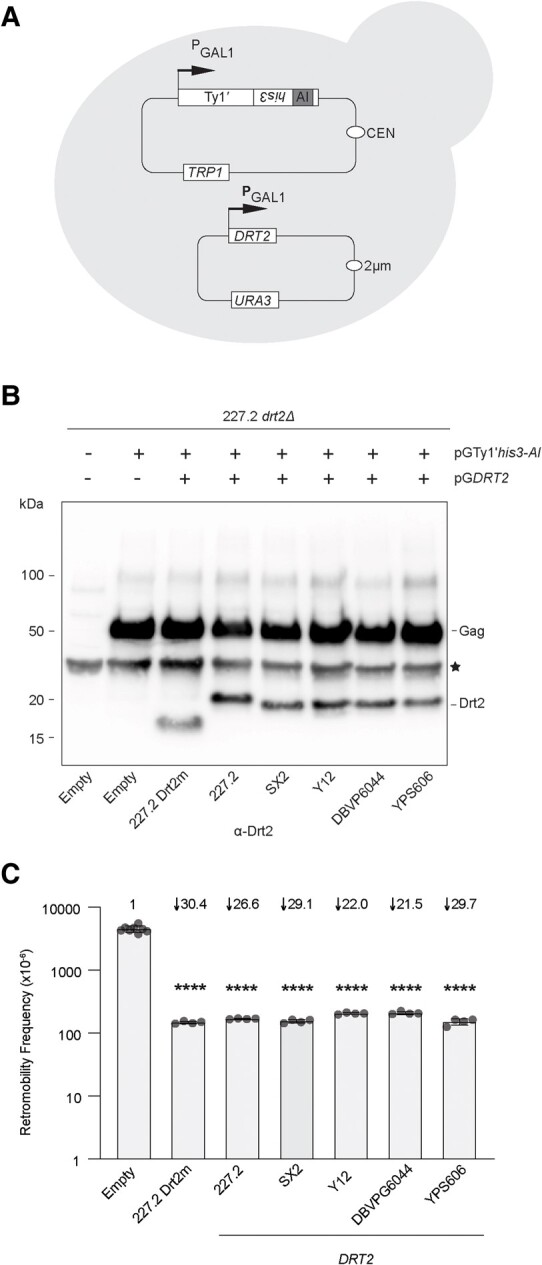
Multiple *DRT2* alleles restrict Ty1′ retromobility. A) Scheme used to determine Ty1′ retromobility in strain 227.2 *drt2Δ*. Shown is a full-length Ty1′ reporter element under control of the inducible *GAL1* promoter (PGAL1) and marked with *his3-AI*. A retromobility event restores histidine prototrophy. The arrow represents the transcription start site. The plasmid contains a low-copy centromere replication sequence (CEN) and the *TRP1* gene for plasmid selection in yeast. *DRT2* variant alleles, including the truncated 227.2 Drt2m (CA-CTD only), expressed from the *GAL1* promoter are present on a 2 μ multicopy plasmid with the *URA3* gene for plasmid selection in yeast. B) Western blot of Ty1′ Gag and Drt2 in strains used to determine the level of retromobility. Whole cell extracts prepared from galactose-induced cells were probed with α-Drt2 antibody. A black star indicates a nonspecific protein recognized by α-Drt2. Plasmids harbored in each strain are indicated above blot. The label below the graph indicates the *DRT2* variant allele or the empty multicopy plasmid control. All strains are 227.2 *drt2Δ* derivatives. C) Retromobility frequency of variant *DRT2* alleles from independent strains. Cells were grown under galactose-inducing conditions, and frequency of cells able to grow on selective media was determined. All strains are 227.2 *drt2Δ* derivatives. Statistical significance was measured with Welch's *t*-test comparison of WT with *drt2Δ* strains. ****P < 0.0001 with exact values reported in supplementary table S4, Supplementary Material online.

While the *DRT2* ORF is conserved in many strains, *DRT2* alleles vary in their amino acid sequence. To survey the functional impact of this sequence variation, we chose 5 representative alleles from the breadth of *DRT2* sequence as well as strain diversity and tested their ability to function as restriction factors against Ty1′ in the 227.2 *drt2Δ* genetic background using an established overexpression approach (Fig. 2, supplementary fig. S4, Supplementary Material online) (Nishida et al. 2015; Tucker et al. 2015; Cottee et al. 2021). This assay allows us to test the effect of *DRT2* alleles on Ty1′ retromobility from diverse strains where other Ty and host factor content could influence the results. To systematically compare restriction potency of *DRT2* genes from strains 227.2, SX2, Y12, DBVP6044, and YPS606, allelic *DRT2* ORFs were expressed from the *GAL1* promoter on a multicopy plasmid (pG*DRT2* plasmids pBDG1758 to 1763; supplementary table S3, Supplementary Material online). A minimal 227.2 *DRT2* sequence containing only the CA-CTD (Drt2m) predicted to be sufficient for retromobility restriction based on previous studies of truncated p22 (Nishida et al. 2015; Cottee et al. 2021) was also analyzed for comparison (supplementary fig. S4B, Supplementary Material online). The *DRT2* expression constructs or empty vector were introduced into strain 227.2 *drt2Δ* containing a *GAL1*-promoted Ty1′*his3-AI* element on a separate plasmid (Fig. 5A). Western blot analysis using α-Drt2 of strains coexpressing Ty1′*his3-AI* and *DRT2* showed similar levels of Gag and Drt2 proteins (Fig. 5B). α-Drt2 also recognized Ty1′ Gag as Drt2 and Gag share considerable sequence identity. Retromobility was measured in these strains and compared with that of a strain containing Ty1′*his3-AI* and an empty vector control. When *DRT2* derived from strains 227.2 (pBDG1759), SX2 (pBDG1760), Y12 (pBDG1763), DBVP6044 (pBDG1762), and YPS606 (pBDG1761) was coexpressed with pGTy1′*his3-AI*, Ty1′ retromobility decreased by ∼22- to 30-fold, and the expression of 227.2 Drt2m (pBDG1758) resulted in a comparable level of restriction at 30.4-fold (Fig. 5C). Our results demonstrate that variant *DRT2* alleles from diverse strains are capable of restricting Ty1′ retromobility.

### Drt2 Associates with Ty1′ VLPs

**Fig. 6.**
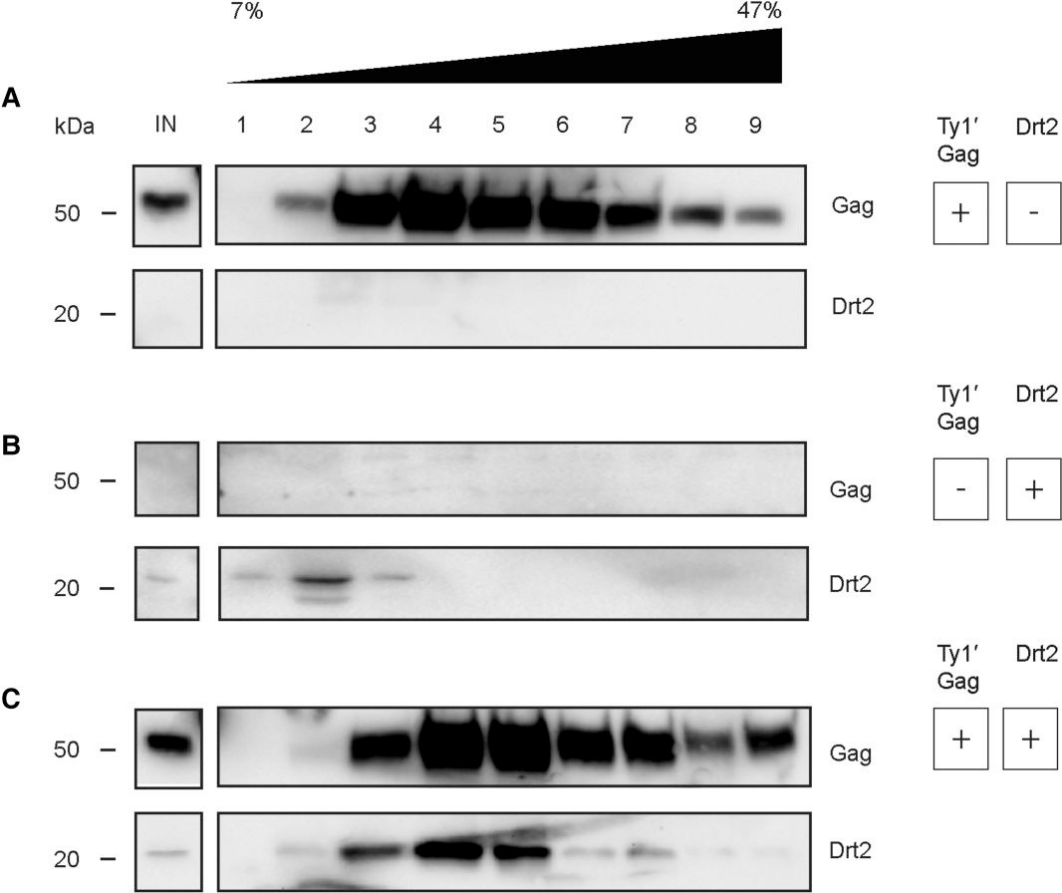
Drt2 associates with Ty1′ VLPs. Whole cell protein extracts from galactose-induced cultures were separated by velocity sedimentation over a continuous 7% to 47% sucrose gradient. Protein input (IN) from each gradient and fractions collected across the gradient are denoted at the top, with 1 containing the lowest and 7 containing the highest sucrose concentration. Representative western blots of ≥3 replicates are shown. α-Ty1′ primary antibody was used to detect Ty1′ Gag, and α-6xhis tag primary antibody was used to detect Drt2. Molecular weight markers are indicated alongside the blots. A) 227.2 *drt2Δ* strain containing pGTy1′*his3-AI* and empty vector plasmid shows Ty1′ Gag sedimentation in the absence of Drt2. B) 227.2 *drt2Δ* strain containing empty vector plasmid and pG*DRT2* shows Drt2 sedimentation in the absence of Ty1′ proteins. C) 227.2 *drt2Δ* strain containing pGTy1′*his3-AI* and pG*DRT2* shows Drt2 sedimentation in the presence of Ty1′ proteins.

Given the sequence similarities between the Ty1′ *GAG* CA-CTD and *DRT2* and previous work on the molecular basis of Ty1c restriction (Cottee et al. 2021), we hypothesized that the mechanism of *DRT2* restriction is mediated through interactions with Ty1′ Gag in assembling VLPs. To determine if Drt2 associates with Ty1′ VLPs or assembly intermediates as was shown for p22 and Ty1c Gag (Saha et al. 2015; Tucker et al. 2015; Cottee et al. 2021), we analyzed the sedimentation pattern of Drt2 and Ty1′ Gag in whole cell extracts prepared from strain 227.2 *drt2Δ* expressing pGTy1′*his3-AI*, pG*DRT2*, or empty vector. Three strains were constructed: one containing pGTy1′*his3-AI* plus empty vector, a second containing empty vector plus pG*DRT2*, and a third containing pGTy1′*his3-AI* plus pG*DRT2*. Cell extracts were sedimented through a 7% to 47% continuous sucrose gradient in the presence and absence of Ty1′ Gag, followed by western blotting of individual fractions (Fig. 6). This approach minimizes bias of Gag oligomeric state as VLP assembly can be monitored in fractionated whole cell extracts (Saha et al. 2015; Tucker et al. 2015; Cottee et al. 2021). Larger assembly complexes and VLPs enter the gradient and fractionate in denser sucrose fractions, whereas smaller complexes and soluble proteins are present at the top of the gradient in less dense fractions. When pGTy1′*his3-AI* was expressed alone, we observed a distribution across the gradient characteristic of VLPs with Gag peaking in fractions 3 to 6 (Fig. 6A) (Saha et al. 2015; Cottee et al. 2021). Drt2 expressed alone sedimented near the top of the gradient primarily in fraction 2, suggesting that it cannot form VLP-sized complexes (Fig. 6B). Coexpression of both Ty1′*his3-AI* and Drt2 resulted in a similar sedimentation pattern for Gag, but Drt2 now cosedimented with Gag (Fig. 6C). The shift in sedimentation pattern of Drt2 when coexpressed with Ty1′*his3-AI* suggests there is an interaction between Drt2 and larger complexes or Ty1′ VLPs and that Drt2 resembles Ty1c p22 in its sedimentation properties and mode of action.

### Capacity of Drt2 to Function as a Gag CA-CTD

**Fig. 7.**
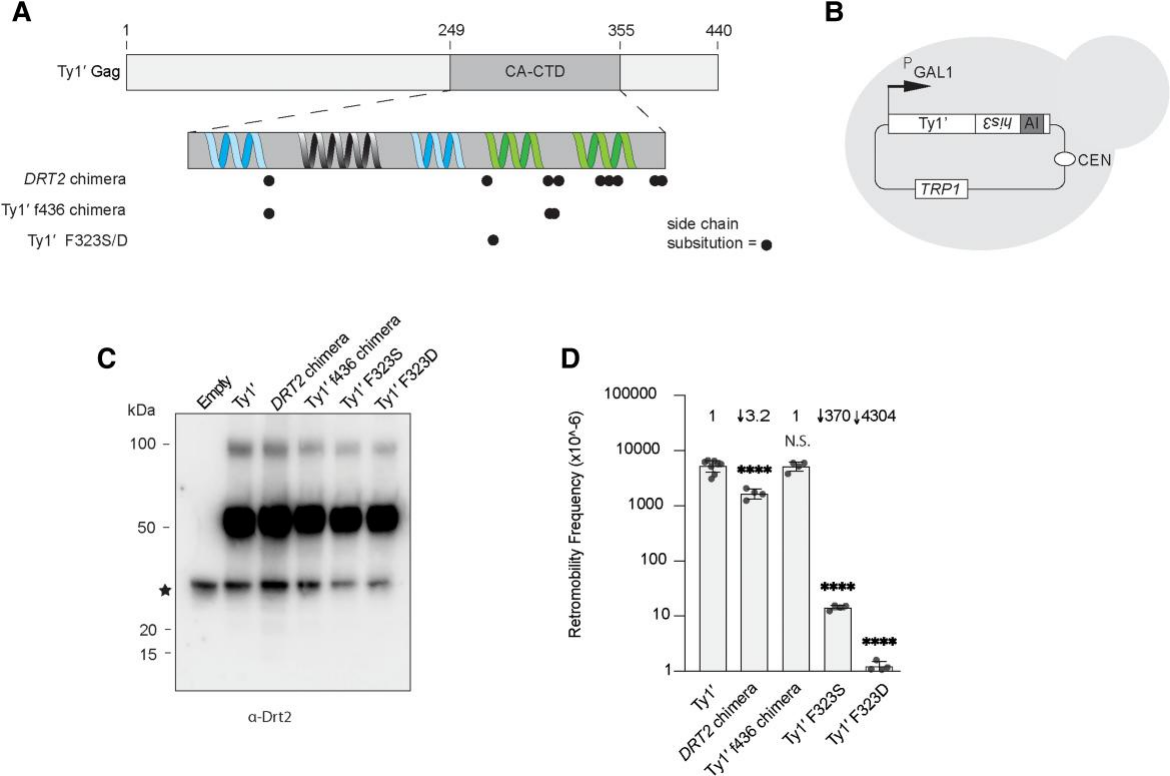
*DRT2* can function as a Ty1′ *GAG* CA-CTD. A) Schematic of constructs used to determine chimeric element retromobility. Predicted alpha helices in the CA-CTD region are indicated with those participating in dimer-1 formation (blue) and those participating in dimer-2 formation (green). The location of side chain substitutions relative to Ty1′ Gag CA-CTD (from S288c YBLWTy1-1) is indicated with circles. Substitutions are described in supplementary fig. S4, Supplementary Material online. B) Schematic to determine retromobility of Ty1′-Gag CA-CTD variants in strain 227.2 *drt2Δ*. WT, chimeric, or mutant Ty1′ reporter elements are under the control of the *GAL1* promoter and contain *his3-AI* (PGTy1′). C) Western analysis of whole cell extracts from cells induced for expression to detect Gag protein with an α-Drt2 primary antibody. Black star indicates a nonspecific band. D) Quantitative retromobility for WT, chimeric, and mutant Ty1′ elements. Bars represent the average ≥4 independent measurements with each measurement shown as a circle. Standard deviation represented by error bars. Fold change compared with WT shown above bars. Statistical significance was measured with Welch's *t*-test comparison of YBLWTy1-1 with each mutant construct. N.S., not significant. ****P < 0.0001 with exact values reported in supplementary table S4, Supplementary Material online.

*DRT2* likely evolved from a functional Ty1′ element and has lost its ancestral function as a complete Gag protein. In the exapted state, *DRT2* encodes a restriction factor of Ty1′ through domestication of the Gag CA-CTD. To test Drt2's potential ancestral functionality as a Gag CA-CTD, we generated several pGTy1′*his3-AI* reporter plasmids containing different CA-CTD sequences. In addition to the *DRT2* CA-CTD from 227.2, we chose a CA-CTD sequence from full-length chromosomal Ty1′ element f436 in strain UWOPS05-787.3 (Czaja et al. 2020). We also analyzed Ty1′ elements containing single amino acid side chain substitutions F323S and F323D in the CA-CTD dimer-2 region that are predicted to abrogate Ty1′ retromobility based on previous analyses of Ty1c Gag (Fig. 7A, supplementary fig. S5, Supplementary Material online) (Cottee et al. 2021). The plasmids were introduced into strain 227.2 *drt2Δ*, and the level of Gag and the Ty1′ retromobility frequency was determined (Fig. 7B). Strains grown under inducing conditions had similar levels of Gag as detected by western blotting (Fig. 7C). Relative to a WT Ty1′ (YBLWTy1-1), quantitative retromobility measurements showed a modest 3-fold decrease for the *DRT2* chimera and no significant difference for the Ty1′ f436 chimera, whereas the CA-CTD dimer-2 mutants F323S and F323D decreased mobility ∼400- and ∼4,000-fold, respectively (Fig. 7D). Together, our results suggest that the Drt2 CA-CTD-like sequence retains considerable functionality as a Gag CA-CTD after domestication. Additionally, the decreased mobility without markedly reducing Gag protein levels of the F323S and F323D mutants supports the conservation of Ty1′ Gag dimer-2 and its role in VLP assembly (Cottee et al. 2021).

### Ty1′ Does Not Display Self-Encoded CNC

**Fig. 8.**
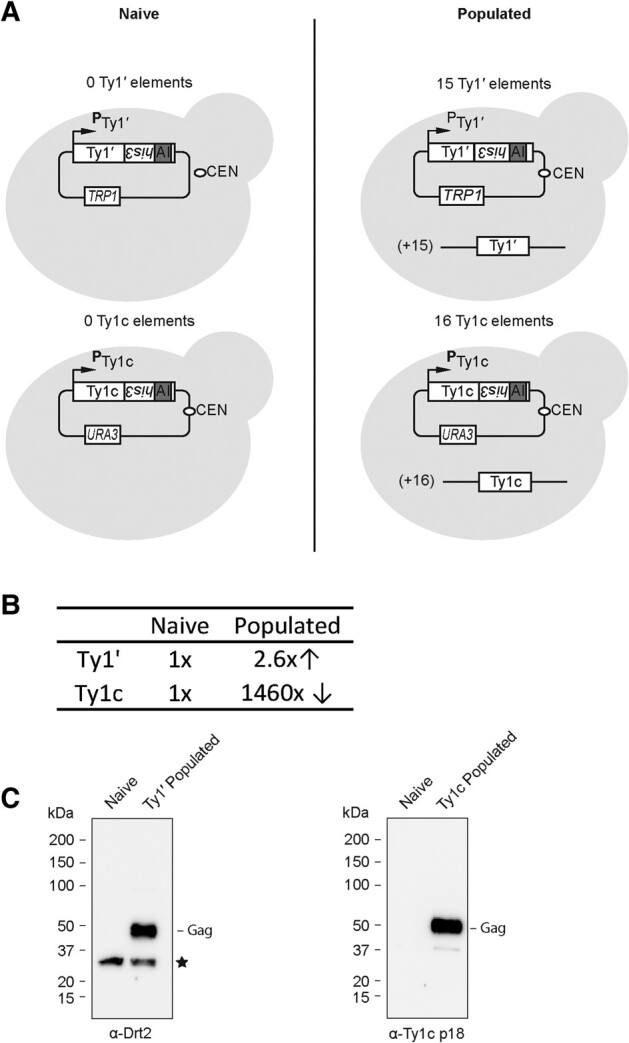
Ty1′ lacks self-encoded CNC. The 227.2 *drt2Δ* strains were populated with either Ty1′ (YBLWTy1-1) or Ty1c (Ty1-H3) elements. Ty1′ and Ty1c copy number was estimated by southern blotting (supplementary fig. S6, Supplementary Material online). A) Retromobility of pGTy1′*his3-AI* and pGTy1c*his3-AI* was determined in the naïve strains in triplicate. B) Retromobility of pGTy1′*his3-AI* and pGTy1c*his3-AI* was measured in triplicate in the populated strains. Fold change in retromobility of populated versus naïve strains is indicated. Retromobility measurements and statistics are reported in supplementary table S4, Supplementary Material online. C) Western blot analysis of whole cell extracts from strains induced for expression was used to detect the level of Gag in populated strains. Ty1′ Gag was detected with α-Drt2 (black star indicates nonspecific band). Ty1c Gag was detected with α-Ty1c p18.

To determine if Ty1′ confers self-encoded CNC, strain 227.2 *drt2Δ* was populated with Ty1′ or Ty1c elements obtained by transposition induction of a pGTy1′ or pGTy1c plasmid, respectively (Boeke et al. 1985; Garfinkel et al. 2003). The resulting strains contained ∼15 Ty1′ and ∼16 Ty1c elements as estimated by Southern blot analysis (supplementary fig. S6, Supplementary Material online). Ty1′*his3-AI* and Ty1c*his3-AI* reporter plasmids were introduced into the parental and populated strains, and the level of retromobility was determined. Ty1′*his3-AI* mobility increased 2.6-fold in the Ty1′ populated strain when compared with the naive strain, while retromobility of the Ty1c*his3-AI* mobility decreased 1,460-fold in the Ty1c populated strain when compared with the naive strain (Fig. 8A and B). In addition, using primary antibody α-Ty1c p18 (Gag residues 249 to 401) for Ty1c and α-Drt2 for Ty1′, we confirmed that the insertion of chromosomal elements in the populated strains resulted in the expression of Gag (Fig. 8C). Reduction in retromobility of Ty1c with increasing copy number supports several previous studies (Garfinkel et al. 2003; Garfinkel et al. 2005; Saha et al. 2015; Tucker et al. 2015; Czaja et al. 2020) and demonstrates that the 227.2 *drt2Δ* background has the capacity to permit robust CNC. In contrast, we detected an increase in Ty1′ retromobility in a strain populated with additional Ty1′ elements, which suggests that Ty1′ does not undergo self-encoded CNC through production of a p22-like protein as copy number increases.

## Discussion

In this study, we combine phylogenomic and molecular genetic approaches to characterize the evolutionary history and functionality of a novel TE restriction factor coopted from a family of active TEs. Our work demonstrates that *DRT2* is present in many diverse clades of *S. cerevisiae*, including the most ancestral Chinese lineages, and displays extensive structural variation in the relic 2 region. The data support the idea that *DRT2* was endogenized as a restriction factor from an ancestral Ty1′ element prior to the divergence of extant *S. cerevisiae* lineages. Domestication of the Ty1′ Gag CA-CTD provides a unique example of a host repurposing a TE protein as a defense mechanism against the TEs within a highly dynamic region of the *Saccharomyces* genome.

Our data extend previous work characterizing truncated Ty1′ relics that are at a fixed locus and present in multiple strains. Relic 2 was initially described as containing the solo LTR, and *GAG* sequence was closely related to Ty1′ and detected in 15 of the 41 strains surveyed (Bleykasten-Grosshans et al. 2013). Our ML phylogenetic tree based on the 1 kb sequences on either side of the relic 2 locus broadly recapitulates host strain phylogenies based on whole genome sequencing data (Fig. 2) (Peter et al. 2018; O'Donnell et al. 2023). Interestingly, the Ty integration mechanisms used to target Ty1, Ty2, Ty3, and Ty4 insertions to genes transcribed by RNA Pol III (Bridier-Nahmias et al. 2015; Patterson et al. 2019; Chen et al. 2023) make relic 2 a hot-spot for Ty activity. Indeed, high-resolution genomic sequencing demonstrates a large amount of structural variation due to Ty activity at the relic 2 locus, including additional full-length element insertions and solo LTRs. The Ty1′ insertion is maintained at the relic 2 locus but lacks the sequence capacity to encode full-length Gag due to truncation at the 5′ and 3′ ends (Czaja et al. 2020). This type of degradation of the coding sequence of a full-length element is uncommon in *S. cerevisiae* as elements are typically lost by removal of the coding sequence via homologous recombination between LTR sequences bracketing the element (Jordan and McDonald 1999; Moore et al. 2004; Carr et al. 2012). Remarkably, relic 2 retains the capacity to encode a p22-like protein containing the entirety of the structured CA-CTD region (Fig. 1), and this ORF is present in many diverse strains (Fig. 2). *DRT2*'s presence in the deep CHN-IX, CHN-II, and CHN-I clades suggests it originated early in the evolutionary history of *S. cerevisiae* (Peter et al. 2018; O'Donnell et al. 2023). In our data set, *DRT2* loss events were also detected in HN1, PD27A, and the European wine/Mediterranean oak clade. Wine strains have unique specializations to their environment, such as horizontally transferred oligopeptide transporter *FOT* genes and at least 37 highly divergent genes, but none present an obvious connection to *DRT2* loss (Coi et al. 2017; Marsit et al. 2017). It remains unclear why these strains lost *DRT2* at the relic 2 locus or elsewhere in the genome. Interestingly, there is an apparent gene conversion of *DRT2* in strains BCN and CNT that now contains the homologous region of a different Ty1 subfamily (Ty101). Although further studies are required to determine if the recombinant gene is capable of restriction factor function, the presence of *DRT2* gene conversions raises the possibility that a cassette-like mechanism can be used to generate variation in restriction factor specificity, which is conceptually similar to mating type switching in *S. cerevisiae* (Klar and Fogel 1979; Strathern and Herskowitz 1979).

Our findings establish the restriction factor functionality of *DRT2*. Under conditions that mimic exposure of a strain with a Ty1′ naive genome to Ty1′ proteins poised for insertion events, we show that *DRT2* restricts the ability of Ty1′ to undergo retrotransposition in strains 227.2 and SX6 (Fig. 3). In conjunction with our survey of *DRT2* variants (Fig. 5), it is likely that all strains with *DRT2* are less susceptible to Ty1′ transposition events. Over evolutionary time scales, this restriction could result in a higher fitness of the host due to lower chances of deleterious effects associated with high TE burden (Scheifele et al. 2009). The inability to detect endogenous Drt2 by western blotting is likely due to the sensitivity of the polyclonal primary antibody, α-Drt2, against Drt2 used in this study (supplementary fig. S2, Supplementary Material online). Our data suggest that *DRT2* transcription initiates at a Ty1i-like internal site rather than initiation at the site used for transcription of full-length Ty1′ RNA (Fig. 4). The Ty1i start site of Ty1c gives rise to a transcript that encodes the p22 restriction factor containing the CA-CTD but not full-length Gag (Saha et al. 2015). Therefore, the host may have evolved transcription signals to express *DRT2* rather than full-length Ty1′ RNA. Further studies are required to evaluate if *DRT2* transcription regulation responds to stress conditions that increase full-length Ty1 transcription and the role the mediator complex plays in the choice of initiation sites (Morillon et al. 2002; Salinero et al. 2018).

We show that ectopic expression of variant *DRT2* alleles confers similar restriction factor activity against Ty1′*his3-AI*, suggesting that this function may be present in all strains containing *DRT2*. Given the complexity of the relic 2 region in different strains, ectopic expression of *DRT2* allows for direct comparison of restriction strength in an isogenic background. Consistent with previous studies (Nishida et al. 2015; Cottee et al. 2021), we show that the minimal *DRT2* construct containing only the predicted structured regions of the protein (Drt2m) is sufficient for restriction (Fig. 5). However, *DRT2* restriction of Ty1′ in the ectopic expression studies is much lower than that observed with p22 restriction of Ty1c (Nishida et al. 2015; Cottee et al. 2021). One possible explanation for this difference is that *DRT2* is present in the genome and restricts Ty1′ regardless of the current genomic copy number, while p22 appears to take effect only after a number of elements have populated the genome to prevent rampant Ty1c insertion events (Garfinkel et al. 2003; Garfinkel et al. 2005; Tucker et al. 2015). Further studies are necessary to determine if *DRT2* is capable of restricting other Ty families and what sequences determine restriction specificity. Together, our results demonstrate that *DRT2* restricts Ty1′ retromobility and is actively transcribed in independent strains.

Understanding how restriction factors inhibit virus-like particle assembly informs TE biology. In the context of full-length Ty1c Gag, CA-CTD–CA-CTD interactions are necessary for VLP assembly (Cottee et al. 2021); however, the p22 restriction factor lacks the CA-NTD. The altered stoichiometry of CA-NTD–CA-CTD and CA-CTD–CA-CTD interactions resulting from excess CA-CTD protomers disrupt the interactions with full-length Gag required to assemble functional VLPs (Cottee et al. 2021). For Ty1c, the interaction between the p22 restriction factor and full-length Gag is observed during velocity sedimentation assays where Ty1c Gag and p22 associate and alter the size distribution of assembly intermediates (Saha et al. 2015; Tucker et al. 2015). We provide evidence that Drt2 associates with Ty1′ VLPs through the shift of Drt2 into higher molecular weight fractions when coexpressed with Ty1′ Gag and compared with Drt2's sedimentation profile when expressed alone (Fig. 6). The minor shift of Ty1′ Gag sedimentation when coexpressed with Drt2 is less than p22-induced Ty1c sedimentation and likely reflects the lower level of Ty1′ restriction mediated by Drt2. More detailed studies of the interaction between Drt2 and Ty1′ are required to elucidate how these proteins associate; however, our data suggest an association between Drt2 and assembling Ty1′ VLPs that disrupts productive VLP assembly.

We hypothesize that *DRT2* originated from the CA-CTD region of an ancient full-length Ty1′ *GAG* and that the extant *DRT2* sequence we observe may have had CA-CTD function in the context of a full-length element. Here, we demonstrate that *DRT2* can function as a Gag CA-CTD in the context of a full-length element by creating an active chimeric Ty1′*his3-AI* element containing the *GAG* CA-CTD from *DRT2* in 227.2. The Ty1′-*DRT2* element displays a retromobility frequency ∼3-fold lower than the parental Ty1′ element but is much more active than the Ty1′ F323S/D mutants containing a defective CA-CTD (Fig. 7). Therefore, the CA-CTD sequence of *DRT2* appears to retain the ancestral capacity for productive VLP assembly when placed in the context of a full-length Gag and acts as a restriction factor in its extant state at relic 2. In both roles, the underlying mechanism involves dimerization of the CA-CTD regions of the proteins, but when one of the proteins lacks the other Gag components necessary for VLP assembly, transposition is blocked.

A related question concerning the evolutionary history of *DRT2* is whether Ty1′ full-length elements can self-encode the restriction factor. Ty1c full-length elements produce more inhibitory p22 as copy number increases and results in a large reduction of Ty1c mobility in a genome populated with full-length Ty1c elements (Garfinkel et al. 2003; Saha et al. 2015; Tucker et al. 2015; Ahn et al. 2017; Czaja et al. 2020). In this study, we provide evidence that Ty1′ does not display the same CNC phenomenon as observed with Ty1c. When a strain is populated with ∼15 Ty1′ full-length elements, there is 2.6-fold *increase* in Ty1′ retromobility, whereas a strain populated with ∼16 Ty1c full-length elements results in a 1,460-fold *decrease* in Ty1c mobility (Fig. 8). Consequently, the Ty1′ lack of self-encoded Ty1′ CNC raises questions about the evolution of CNC in the Ty1 family. It appears that Gag CA-CTD–CA-CTD interactions have been exploited for control of retromobility in both *DRT2* repression of Ty1′ and the self-encoded p22 control of Ty1c. Based on the current evidence, it remains unclear if Ty1′ possessed a form of self-encoded p22-like control and lost this mechanism or if *DRT2* domestication was sufficient to relieve the pressure for evolution of self-encoded CNC in the active Ty1′ lineage. Perhaps the loss of RNAi machinery in *S. cerevisiae* drove the evolution of these CA-CTD interaction–based control mechanisms of Ty1c and Ty1′ (Drinnenberg et al. 2009).

Our work also raises the possibility that additional restriction factors of LTR retrotransposons await discovery in *S. cerevisiae*. For example, several strains contain a truncated Ty1′ element termed relic 1 at a different location on chromosome IV (Bleykasten-Grosshans et al. 2013, Czaja et al. 2020). Since relic 1 contains the coding potential for a complete Ty1′ Gag-like protein, a hypothetical relic 1 protein could inhibit Ty1′ VLP assembly. However, further work is necessary to characterize the evolutionary history and functional role of relic 1 in the Ty1′ lifecycle. If *S. cerevisiae* domesticated *DRT2* as a restriction factor, it is plausible that restriction factors from other Ty families may have been domesticated across *Saccharomyces*. Ty2-5 all have the capacity to form VLPs, and Ty3 VLPs and Ty5 VLP proteins have been characterized (Sandmeyer et al. 2015; Irwin and Voytas 2001); thus, inhibiting VLP assembly by truncated Gag proteins through mechanisms similar to *DRT2* inhibition of Ty1′ and p22 inhibition of Ty1c could provide effective restriction factors for these Ty families as well.

TEs and their hosts have coevolved a balance between element movement and host fitness required for long-term success. Here, we present the first example of domestication of a TE protein domain necessary for transposition and its endogenization as a defense factor against TE propagation in the well-studied *Saccharomyces* model. Two examples of TE Gag proteins being repurposed for restriction of LTR retroelements have been documented in other species. Murine leukemia virus (MLV) is a well-studied retrovirus that causes cancer in mice through insertion events that alter oncogene expression (De Ravin et al. 2014; LaFave et al. 2014). Fv1 is a restriction factor of MLV derived from the *GAG* gene of an endogenized retrovirus and, like *DRT2*, acts through interactions with the MLV capsid during infection (Jolicoeur and Rassart 1980; Best et al. 1996). Fv1 has 2 alleles that have a specificity of restriction for certain subtypes of MLV (Rowe 1972; Rowe and Hartley 1972), inviting the question of whether *DRT2* has the capacity to restrict elements from other Ty1 subfamilies besides Ty1′ and if there are more endogenized restriction factors specific for other Ty elements. Another example is that of the Jaagsiekte sheep retrovirus (JSRV) which causes lung cancer in sheep through its insertional activity (Armezzani et al. 2014). JSRV is restricted by an endogenized JSRV *GAG* gene that blocks assembly through capsid interactions (Murcia et al. 2007; Hofacre and Fan 2010). There are also similar phenomena with non-LTR retroelements where truncated copies of the LINE-1 retrotransposon in humans inhibit LINE-1 mobility (Sokolowski et al. 2017). Truncated LINE-1 ORF1 proteins contain the N-terminal domain and the coiled-coil domain and retain the ability to form trimers with full-length ORF1. The incorporation of truncated ORF1 proteins lacking the RNA recognition motif and the C-terminal domain into higher order structures is responsible for inhibiting LINE-1 mobility. Thus, p22 and Drt2 from budding yeast, as well as truncated ORF1 from humans, utilize a similar strategy to restrict retrotransposon mobility by mediating the assembly of nonfunctional oligomers. The work presented here increases our understanding of endogenized *GAG* sequences and other TE proteins as restriction factors (Rowe 1972; Rowe and Hartley 1972; Jolicoeur and Rassart 1980; Best et al. 1996; Murcia et al. 2007; Hofacre and Fan 2010; Sokolowski et al. 2017). Further, these endogenization events can occur at the species level and the function of these new genes may be related to TE activity rather than novel cellular functions.

## Materials and Methods

### DNA Preparation and Genome Sequencing

To prepare DNA for PacBio sequencing, single colonies of strains HN6alpha, HN9alpha, BJ20alpha, HN14alpha, SD1alpha, and HLJa/alpha were separately inoculated in 7 mL of yeast extract–peptone–dextrose (YPD) liquid broth and cultured for ∼24 h at 30 °C. DNA was isolated using a modification of the Wizard Genomic DNA Purification Kit (Promega cat. #A1125) as previously described (Czaja et al. 2020). DNA was sheared and size selected (<10 kb) using a Covaris g-Tube prior to multiplex adapter ligation and PacBio library preparation using the SMRTbell Express Template Prep Kit (Pacific Biosciences). Multiplex PacBio sequencing of all samples was performed simultaneously with the Sequel II instrument (Sequencing Kit v2.1). Demultiplexed sequences for each strain were submitted to Sequence Read Archive (SRA) under project PRJNA774776.

### Preprocessing of Long-Read Sequencing Data and Local Assembly for the *DRT2* Region

PacBio and ONT reads from the current and previous studies were downloaded from SRA in different formats depending on the original submission (.bax.h5, .bam, or .fastq). For PacBio reads downloaded from SRA in raw .bax.h5 files, .bax.h5 files were first converted to .bam files with bax2bam (v0.0.9, https://anaconda.org/bioconda/bax2bam) and then to .fasta files using bam2fastx (v1.3.0, https://github.com/PacificBiosciences/bam2fastx). For PacBio reads downloaded from SRA in .bam format, SAMtools fasta (v1.12) (Li et al. 2009) with default parameters was used to convert .bam to .fasta format. For PacBio and ONT data downloaded in .fastq format, seqtk (v1.3, seq -a, https://github.com/lh3/seqtk) was used to convert .fastq to .fasta format. Subsequently, raw reads in .fasta format were mapped to a complete genome assembly of *S. cerevisiae* strain UWOPS05-227.2 (GenBank: GCA_902192315.1) (Czaja et al. 2020) using minimap2 (v2.18, -ax map-pb) (Li 2018). Supplementary and low-quality alignments were subsequently filtered out by applying samtools view (v1.12) (Li et al. 2009) using parameters “-F 2304 -q 30.” All reads that mapped to the relic 2 locus (defined by the outer limits of the 2 flanking tRNAs: GlyGCC on the 5′ side and SerAGA on the 3′ side) with the exception of reads fully contained within the relic 2 locus were extracted using bedtools intersect (v2.30.0) (Quinlan and Hall 2010) and used for input to perform local assembly. Two genome assemblers were used for local assembly, Flye (v2.8.3) (Kolmogorov et al. 2019) and wtdbg2 (v2.5) (Ruan and Li 2020). Flye assemblies were performed with the “--pacbio-raw” parameter preset. Wtdbg2 was performed with parameters “-q -x rs -AS 1 -g 20k -L 3000” for PacBio sequences and “-q -x ont -AS 1 -g 30k” for ONT sequences.

### Quality Control of Local Assemblies

To check for consistency in read depth and distribution across local assemblies, reads that were used as input for Flye and wtdbg2 assembly were mapped back to the local assemblies with minimap2 (v2.18) (Li 2018) using parameters “-ax map-pb” for PacBio data and “-ax map-ont” for ONT data. Supplementary and low-quality alignments were then excluded by applying samtools view (v1.12) (Li et al. 2009) using parameters “-F 2304 -q 30.” The command line version of IGV (v2.9.5) (Robinson et al. 2011) was then used to generate visualizations of the read-to-local assembly coverage profiles before and after filtering nonprimary alignments. Discrepancies in coverage profiles between nonfiltered and filtered alignments were used to detect local assemblies with potential errors (e.g. collapsed duplications or artifactual duplications) and were classified as “fail” in our quality control process. To evaluate if local assemblies completely spanned the relic 2 locus, Mummer (v3.23) (Kurtz et al. 2004) alignments were performed comparing local assemblies to the relic 2 region from UWOPS05-227.2 (CABIKC010000001.1:521238-523342), which were then visualized with dotPlotly (https://github.com/tpoorten/dotPlotly). If the local assembly did not have a collinear alignment that spanned both flanking regions of the relic 2 locus in UWOPS05-227.2, the local assembly would also be classified as “fail.” Strains that passed both quality control criteria (read coverage and mummer alignment) for both Flye and wtdbg2 local assemblies were included in subsequent analysis of relic 2 structure and evolution (supplementary table S1, Supplementary Material online). Visual inspection revealed that local assemblies generated with Flye contained fewer base errors than those from wtdbg2, presumably because of the default polishing done by Flye but not wtdbg2, and thus, Flye assemblies were used for the following analysis.

### tRNA and Ty Annotation in Local Assemblies of the Relic 2 Locus

tRNA genes flanking relic 2 were annotated using tRNAscan-SE (v2.0.9, default parameters) (Chan et al. 2021) and used to determine the orientation of the local assembly. Ty elements were annotated using a RepeatMasker-based (https://www.repeatmasker.org/) pipeline previously described in Czaja et al. (2020). In order to distinguish the bona fide relic 2 Ty1′ insertion from other truncated elements from Ty1 family members that have inserted into the relic 2 locus, multiple sequence alignments for any truncated Ty1 family sequences in the relic 2 locus were generated with MAFFT (Katoh and Standley 2013) with parameter “--auto” and a neighbor joining (NJ) tree was computed with using SeaView (v5.05) (Gouy et al. 2010) with parameters “-build_tree -distance -BioNJ -nogaps -replicates 100.” Bona fide relic 2 Ty1′ insertions form a monophyletic group, with 2 additional clades corresponding to truncated elements with Ty101 or Ty1c sequences. Final Ty annotations in the Relic 2 locus were visualized using the R package gggenomes (v0.9.4.9000, https://github.com/thackl/gggenomes).

### Phylogenetic Analysis of the *DRT2* Flanking Region

To reconstruct the history of the relic 2 locus, 1 kb regions upstream and downstream of the flanking tRNA genes, respectively, were extracted using seqkit (v0.16.1) (Shen et al. 2016). Both flanking regions (excluding the tRNAs and relic 2 insertion) for each strain were concatenated, and a multiple sequence alignment for concatenated sequences was generated with MAFFT (v7.487) (Katoh and Standley 2013) with default parameters. RAxML (v8.2.12, -f a -m GTRGAMMA -# 100 -x 23333 -p 2333 –no-bfgs) (Stamatakis 2014) was then used to reconstruct a ML phylogeny of the concatenated flanking regions. The resulting ML tree was rooted using a clade containing 4 *S. cerevisiae* strains (EM14S01-3B, CEI, BAH, SX2) from deep Chinese lineages (Istace et al. 2017; Peter et al. 2018; Bendixsen et al. 2021) as an outgroup and then visualized with R package ggtree (Yu et al. 2017).

### Molecular Evolutionary Analysis of *DRT2*

*DRT2* ORF sequences based on PacBio or recent ONT data were extracted from local assemblies and were trimmed at the position of the indel at the end of 227.2 *DRT2* (supplementary fig. S6B, Supplementary Material online). Codon-based multiple sequence alignment was performed with PRANK (v170427, -codon -F -njtree) (Loytynoja and Goldman 2008), which was then used to reconstruct a NJ tree using SeaView (v5.0.4, -build_tree -distance -BioNJ) (Gouy et al. 2010). The codon-aligned *DRT2* sequences, along with the tree, were then used to estimate dN, dS, and dN/dS ratios under PAML (v4.9i) (Yang 2007) model M0 using ETE3 (v3.1.2) (Huerta-Cepas et al. 2016).

### Ty1 Sequence Alignment

Ty1′ Gag (Element YBLWTy1-1 SGD ID: S000006808), Ty1c (Ty1-H3 GenBank accession M18706), and Drt2 (UWOPS05-227.2, Czaja et al. 2020) nucleotide sequences were aligned, and identity was calculated with Geneious software using ClustalW alignment, cost matrix IUB, gap open cost 15, and gap extend cost 6.66. Protein alignments were done using Geneious software using Muscle 3.8.425 and similarity calculated with Blosum90, threshold 0.

### Yeast Strains and Plasmids

Standard yeast genetic and microbiological techniques were used in this work (Guthrie and Fink 1991). All strains were derived from UWOPS05-227.2 Y3629 (Cubillos et al. 2009) or SX6 (Wang et al. 2012; Shih and Fay 2021) and listed in supplementary table S2, Supplementary Material online.

*DRT2* was deleted in strain UWOPS05-227.2 through replacement with the nourseothricin resistance gene (*NatMX*). A PCR product containing the *NatMX* cassette flanked by 40 bp of homology to relic 2 UWOPS05-227.2 genomic sequence (homology indicated by underlined bases) using primers GTAGCGCCTGTGCTTCGGTTACTTCTAAAGAAGTCCAAAC GACATGGAGGCCCAGAATACCCTC and GAGTTCATCATCAGAATGATTAGTGTGATTCACCGTAGAT CAGTATAGCGACCAGCATTCACAT was generated from template pNatMX (Goldstein and McCusker 1999). This PCR product was transformed into UWOPS05-227.2 and plated on YPD + 100 μg/mL nourseothricin (clonNAT, GoldBio cat. #96746). Single colonies were isolated, and gDNA was extracted and validated for *NatMX* cassette integration at relic 2 with PCR using primers GCACACTAACAGGTAGATTG and GCCGTGTCGTCAAGAGTGG.

An ∼1,100 bp PCR fragment containing the *flo8-G427A* mutation from the S288c reference strain was subcloned into the URA3-based integrating plasmid pRS406 to generate pBDG1705. HindIII or SalI digestion of pBDG1705 generated a linear fragment for the integration at *FLO8* and transplacement of *flo8-G427A*. The *flo8-G427A* mutation was introduced into strain UWOPS05-227.2 using 2-step allele transplacement with integrating plasmid pBDG1705.

The 5′ relic 2 structure in strain SX6 was assessed, and *DRT2*'s presence was confirmed with PCR using primers GCACACTAACAGGTAGATTG and CCGAAGCACAGTCGCTACC. The *his3-ΔKanMX* deletion was introduced by microhomologous recombination using a PCR fragment generated with primers ATGACAGAGCARAAAGCCCTAGTAAAGCGTATYACAAATGCGTACGCTGCAGGTCGACGGATCC and YTACATAAGAACACCTTTGGTGGAGGGAACATCGTTGGTAATCGATGAATTCGAGCTCGTTTAA (*HIS3* sequence is underlined) and pKanMX (Wach et al. 1994; Goldstein and McCusker 1999) as a template. The *trp1-hisG* mutation was introduced using the universal gene-blaster pNKY1009 as described previously (Alani and Kleckner 1987). *DRT2* was deleted in SX6 using the same method as described above for strain UWOPS05-227.2.

pBDG1696 (pGTy1′/*TRP1* Cen) was generated from a pGAL1 expression plasmid (pBDG1665) containing the *TRP1* Cen vector pRS404 (Sikorski and Hieter 1989) and the *GAL1* promoter fragment (EcoRI-XhoI) (Boeke et al. 1985) but lacking sites for SmaI, BamHI, SpeI, XbaI, and NotI. pBDG1665 digested with XhoI and ApaI was subjected to HiFi DNA assembly (New England Biolabs, Ipswich MA) using PCR products containing Ty1′ nucleotides 234 to 2,204 (GalTy1priXhoF: CAGTTTGTATTACTTCTTATTCCTCGAGGAGAACTTCTAGTG and TyRP1: CATTGATAGTCAATAGCACTAGACC, S288c genomic clone pBDG1631, containing the full-length Ty1′ element YBLWTy1-1, www.yeastgenome.org, SGD ID: S000006808 as template) and Ty1′ nucleotides 2,180 to 5,915 (RP1F: GGTCTAGTGCTATTGACTATCAATG and DS3jHFR: CAATTCGCCCTATAGTGGGTACCGGGCCCACATGTATGAAACTGGGA, S288c genomic DNA as template). Also, the DNA segments contained sequences that overlap with the pBDG1665 vector, and an XhoI restriction site was introduced into the GalTy1priXhoF primer. HiFi DNA assembly was used to generate pBDG1697 (pTy1′*his3-AI*/*TRP1* Cen) from pBDG1696 using *his3-AI* (Curcio and Garfinkel 1991) gBlocks (Integrated DNA Technologies, Coraville, IA) that contain flanking sequences overlapping the StyI and ApaI restriction sites present in pBDG1696. HiFi DNA assembly reactions were performed using conditions recommended by the supplier. Plasmids were validated by restriction enzyme analysis and DNA sequencing.

pBDG1785 was constructed by removing the *GAL1* promoter of pGTy1′*his3-AI* and replacing it with the promoter region of YBLWTy1-1 (SGD ID S000006808) from strain S288C. Restriction fragments containing the *GAL1* promoter (EcoRI-XhoI), the pRS424 vector backbone (EcoRI-ApaI) (Sikorski and Hieter 1989), pBDG1696 (XhoI-ApaI), PCR product using primers GGAACAAAAGCTGGAGCTCCGATTCCCTTTTGTAGA and TGGGTGCCCGTATACTGGCCAGCCAGAAATATTGGC amplifying the 5′ LTR and a section of *GAG* from S288C genomic DNA, and pBDG1697 fragment (BbvCI-SacII) were assembled using HiFi assembly.

pBDG1758 to 1763 were constructed by cloning gBlocks (Integrated DNA Technologies, Coraville, IA) *DRT2* CDS from different strains with a C-terminal 6xHis tag (more detail in supplementary table S3, Supplementary Material online) using HiFi DNA assembly. pBDG1743 was constructed by cloning into pSP70 digested with HindIII a PCR product from the 227.2 genomic DNA using primers CATCGATGAATTCAAGCTTGACTATACGGGTATCATG and TAGAACTCGAGCAGCTGAGAGAGTTATTAGATGTGG using HiFi assembly. pBDG1814 was constructed using quick change PCR of pBDG1697. pBDG1697 was digested with BbvcI and NruI and 2 PCR products from pBDG1697 using GGTAATTCATTTCCTGATTCA + GCGTAGCGTAAAGATTTGTACTCGC and GCGAGTACAAATCTTTACGCTACGC + CATCGAGGATAGAGTCCTCGC and ligated using HiFi assembly. pBDG1815 was constructed using quick change PCR of pBDG1697. pBDG1697 was digested with BbvcI and NruI and 2 PCR products from pBDG1697 using GGTAATTCATTTCCTGATTCA + GCGTAGCGTAAATCTTTGTACTCGC and GCGAGTACAAAGATTTACGCTACGC + CATCGAGGATAGAGTCCTCGC and ligated using HiFi DNA assembly.

Additional details are available in supplementary table S3, Supplementary Material online. Plasmids were validated by restriction enzyme analysis and DNA sequencing. The Ty1-H3 GenBank accession is M18706.

### Western Blotting

Yeast strains containing *GAL1* expression plasmids were grown at 30 °C overnight in SC-Trp, SC-Ura, or SC-Ura-Trp + 2% raffinose, diluted 1/25 into SC-Trp, SC-Ura, or SC-Ura-Trp + 2% galactose at 22 °C for 48 h. Cells producing endogenous proteins were grown in SC-Trp, SC-Ura, or SC-Ura-Trp + 2% glucose or YEPD for 48 h at 22 °C. Total cell protein was prepared with trichloroacetic acid (TCA) precipitation as previously described (Saha et al. 2015). Briefly, cells were harvested and resuspended in 20% TCA, vortexed with glass beads, and washed in 5% TCA. The precipitate was resuspended in Laemmli buffer and boiled for 10 min. Proteins were separated on 4% to 15% TGX Stain-Free gels (Bio-Rad cat. #4568086). Proteins were transferred to a polyvinylidene difluoride (PVDF) membrane and blotted for Ty1′ Gag or Drt2 with α-Drt2 polyclonal primary antibody (Boster Bio cat. #DZ41275) and for Ty1c Gag with α-Ty1c p18 polyclonal primary antibody (Boster Bio cat. #DZ33975). Precision Plus Kaleidoscope (Bio-Rad cat. #1610395) standard was used for molecular weight estimations. Imaging was performed using WesternBright ECL (Advansta cat. #K-12049-D50) and a Bio-Rad ChemiDoc MP. Total protein was quantified for normalization using 300 s of UV activation and Stain-Free imaging.

### Northern Blotting

Yeast strains were grown in YEPD at 22 °C. Total RNA was isolated using a phenol and phenol/chloroform extraction (Schmitt et al. 1990). Poly(A)^+^ RNA was purified from total RNA extracts with Dynabeads oligo(dT)_25_ (ThermoFisher cat. #61005) per the manufacturer’s instructions. Two μg of poly(A)^+^ RNA was used for *DRT2* probe and 1 μg was used for the actin probe. Poly(A)^+^ RNA was resolved on a 1.2% formaldehyde–agarose gel and transferred to a Hybond-XL (Cytiva cat. #RPN203S). For the *DRT2* probe, pBDG1743 was digested with EcoRV, gel purified, and used as the template for in vitro transcription (IVT). IVT was carried out with the MAXIscript SP6/T7 Transcription Kit (ThermoFisher cat. #AM1322) per the manufacturer's recommendations using SP6 RNA polymerase for the incorporation of [α-^32^P] UTP (800Ci/mmol) (PerkinElmer cat. #NEG007X250UC). Unincorporated nucleotides in the IVT reaction were removed with a ProbeQuant G-50 Micro Column (Cytiva cat. #28903408) per the manufacturer's recommendations. For the actin probe, pBDG1411 was digested with EcoRI, gel purified, and used as the template for IVT. IVT was carried out with the MAXIscript SP6/T7 Transcription Kit (ThermoFisher cat. #AM1322) per the manufacturer's recommendations using T7 RNA polymerase for the incorporation of [α-^32^P] UTP (800Ci/mmol) (PerkinElmer cat. #NEG007X250UC), and unincorporated nucleotides were removed as described above. The probes were added to the hybridization solution, and hybridization, washing, and imaging was performed as previously described (Saha et al. 2015). Phosphorimaging analysis was performed with an Amersham Typhoon 5, according to the manufacturer's suggestions.

### RNA-Seq

The expression of *DRT2* relative to other *S. cerevisiae* genes was determined using public RNA-seq data for strain 227.2 (SAMN02383519) (Lee et al. 2013). The reference genome used in this analysis is a high-quality whole genome assembly of 227.2 (GCA_902192315.1) (Czaja et al. 2020). Sequences of known *S. cerevisiae* genes in S288C were retrieved from SGD and used to annotate genes in the 227.2 genome with minimap2 (-x asm10, v2.22; Li 2018). Quality control and adapter trimming of raw RNA-seq reads were performed with fastp (v0.23.2) using default parameters (Chen et al. 2018). Processed reads were then aligned to the reference genome using HISAT2 (v2.2.1) (Kim et al. 2015) and sorted with SAMtools (v1.15.1) (Li et al. 2009). Reads aligned to each gene were counted with HTSeq-count (v2.0.3, --stranded no) (Anders et al. 2015). Insert sizes were calculated with CollectInsertSizeMetrics function of picard (v3.0.0) (Institute 2019). TPM values were calculated using a custom function in R (v4.2.1).

### Retromobility

The frequency of Ty1′ and Ty1c retromobility was determined using the *his3-AI* indicator gene (Curcio and Garfinkel 1991). For each retromobility measurement, a single colony was suspended in 20 μL water and 5 μL was used to inoculate 4 independent 1 mL cultures. Strains containing pTy1′*his3-AI* (pBDG1785) were grown at 22 °C in SC-Trp + 2% glucose liquid media for 72 h, plated on SC-Trp-His + 2% glucose to determine the number of retromobility events, and plated on SC-Trp + 2% glucose to determine the total number of viable cells, and colonies were counted. Strains containing pTy1*his3-AI* (pBDG633) were grown at 22 °C in SC-Ura + 2% glucose liquid media for 72 h, plated on SC-Ura-His + 2% glucose to determine the number of retromobility events, and plated on SC-Ura + 2% glucose to determine the total number of viable cells, and colonies were counted. Strains containing both pGTy1′*his3-AI* (pBDG1697) and 2 μ plasmids harboring *DRT2* sequences (pBDG1758, pBDG1759, pBDG1760, pBDG1761, pBDG1762, pBDG1763) were grown for 24 h at 30 °C in 1 mL of SC-Ura-Trp + 2% raffinose media, diluted 1:25 1 mL of SC-Ura-Trp + 2% galactose media, grown for 48 h at 22 °C, diluted in water, plated on SC-Ura-Trp-His + 2% glucose to determine the number of retromobility events, and plated on SC-Ura-Trp + 2% glucose to determine total number of viable cells, and colonies were counted. Retromobility was determined in derivatives of strain 227.2 (DG4223). Each retromobility measurement was carried out in triplicate.

### Velocity Cosedimentation

Eight milliliter cultures were grown for 24 h at 30 °C in SC-Ura-Trp + 2% raffinose media, 4 mL diluted 1:25 into 100 mL SC-Ura-Trp + 2% galactose and grown for 48 h at 22 °C, harvested, and lysed with glass beads in 15 mM KCl, 10 mM HEPES-KOH, pH 7.6, 5 mM ethylenediaminetetraacetic acid (EDTA), 100 U/mL RNase inhibitor, 16 μg/mL aprotinin, 16 μg/mL leupeptin, 16 μg/mL pepstatin A, and 2 mM phenylmethylsulfonyl fluoride (PMSF). The cell lysate was clarified by centrifugation at 10,000 × *g*. Approximately 5 mg of total protein was applied to a 7% to 47% continuous sucrose gradient in 15 mM KCl, 10 mM HEPES-KOH, pH 7.6, and 5 mM EDTA and centrifuged at 77,000 × *g* for 3 h at 4 °C. Nine 1.2 mL fractions were collected, and total protein was precipitated by bringing solution to 20% TCA, centrifugation at 10,000 × *g* for 10 min at 4 °C, washed with 5% TCA, resuspended in 1× Laemmli buffer, and boiled for 10 min. Samples were immunoblotted with α-Ty1′ (Boster Bio cat. #DZ33974) to detect Ty1′ Gag and immunoblotted with monoclonal rabbit hexa-histidine antibody clone RM146 (ThermoFisher cat. #MA5-33032) to detect Drt2.

### Populating Strains with Ty1′ and Ty1c Elements

Strain 227.2 *drt2Δ* (DG4302) containing pBDG1696 was transposition-induced on galactose to generate Ty1′ insertions. Briefly, a transformant was passaged 3 times on SC-Trp + 2% galactose plates at 22 °C, replica-plated to YEPD and incubated at 30 °C to allow plasmid segregation, and then replica-plated to FAA to select for cells lacking the *TRP1*-based plasmid (Toyn et al. 2000). Populating DG4302 with Ty1c was performed as described previously (Czaja et al. 2020).

### Southern Blotting

Eight milliliter YEPD cultures were inoculated with single colonies and grown for 48 h at 30 °C, and total DNA was isolated as previously described (Boeke et al. 1985). DNA (∼10 μg) was digested with BglII overnight at 37 °C and separated by electrophoresis on a 0.6% agarose gel for 16 h at 2.3 V/cm. DNA was transferred to Hybond-N membrane (Cytiva RPN1210B) with capillary action and UV-crosslinked according to the supplier's specifications (Spectroline). ^32^P-labeled DNA probes were generated by random-primed DNA synthesis using an Amersham Megaprime DNA labeling kit (Cytiva cat. #RPN1606) with [α-^32^P] dCTP (6000Ci/mmol). For Ty1′, pBDG1697 was digested with BglII and BbvcI and the 942 bp fragment was gel purified and labeled. For Ty1c, pBDG202 was digested with ClaI and HindIII and the 941 bp fragment was gel purified and labeled. Hybridization was carried out at 68 °C overnight in a buffer containing 6× SSC, 5× Denhardt's solution, 0.5% SDS, and 100 μg/mL heat-denatured salmon sperm DNA. Membranes were washed twice with 2× SSC + 0.1% SDS 30 min/wash and twice with 1× SSC + 0.1% SDS, 15 min/wash. All washes were carried out at 68 °C (Czaja et al. 2020). Membranes were wrapped in plastic film and imaged using a Typhoon Phosphorimager, according to the manufacturer's recommendations.

## Supplementary Material

msae050_Supplementary_Data

## Data Availability

Demultiplexed sequences for each strain were submitted to SRA under project PRJNA774776.
