# Supplementary material for: Evolution of a Restriction Factor by Domestication of a Yeast Retrotransposon
Source: Mol Biol Evol. 2024 Mar 5;41(3):msae050. doi: 10.1093/molbev/msae050 (PMC10951436; doi:10.1093/molbev/msae050)
Supplement: msae050_Supplementary_Data [file msae050_supplementary_data.zip › Supplementary_Figures_S1-S6.pdf]

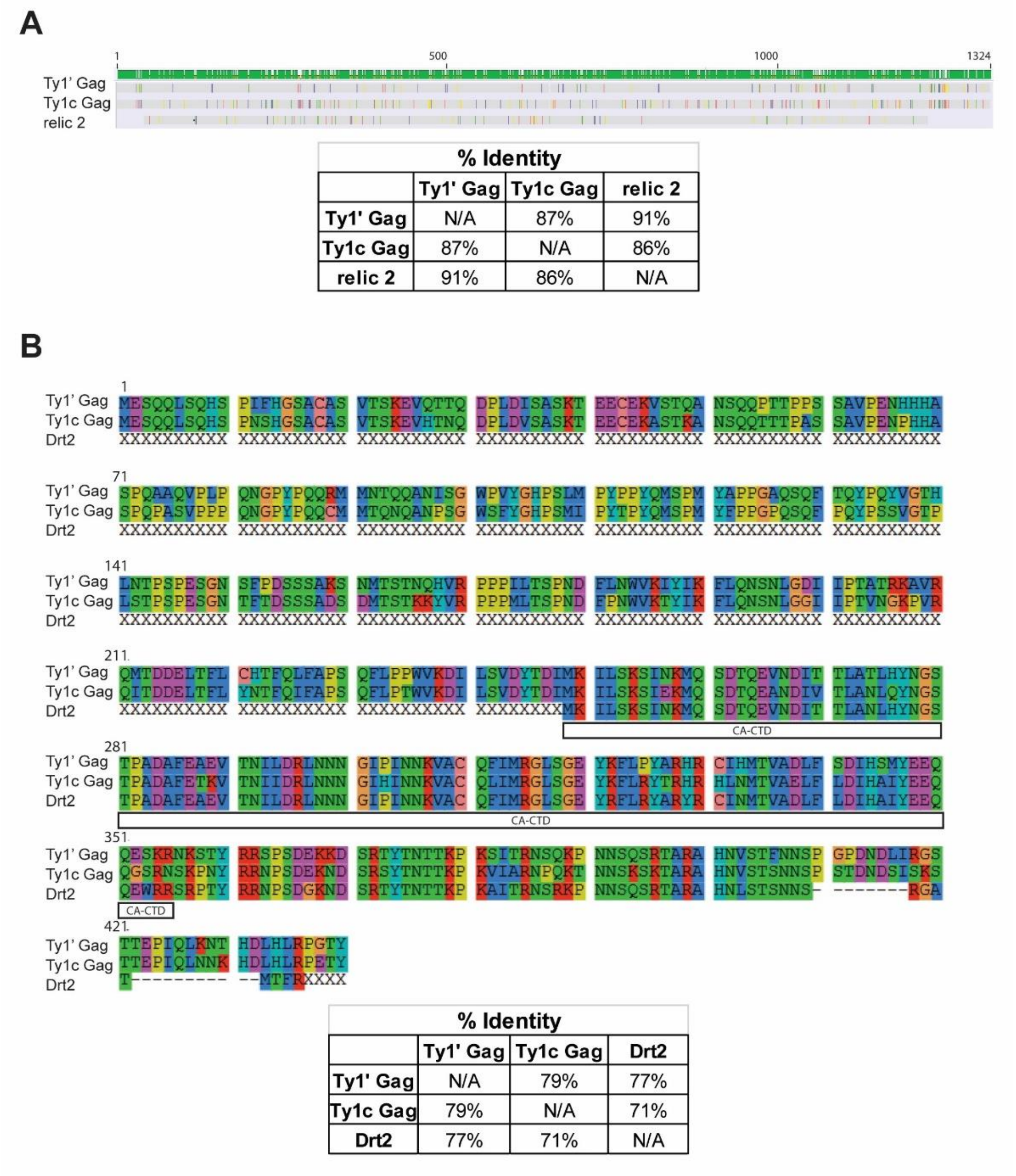

% Identity			
	Ty1' Gag	Ty1c Gag	relic 2
Ty1' Gag	N/A	87%	91%
Ty1c Gag	87%	N/A	86%
relic 2	91%	86%	N/A

% Identity			
	Ty1' Gag	Ty1c Gag	Drt2
Ty1' Gag	N/A	79%	77%
Ty1c Gag	79%	N/A	71%
Drt2	77%	71%	N/A

Supplementary Figure S1. Ty1' Gag, Ty1c Gag, and relic 2 sequence comparison. **A** DNA sequence alignment and percent sequence identity table of Ty1' Gag CDS (element YBLWTy1-1 SGD ID: S000006808), Ty1c Gag CDS (Ty1-H3, GenBank accession M18706), and relic 2 from strain 227.2. Green bar above sequences represents consensus, full height indicates full consensus, half height indicates consensus between 2 sequences, no bar indicates no consensus. Colored lines in each track indicate nucleotide deviation from consensus, A in red, C in blue, G in yellow, T in green. **B** Protein sequence alignment and percent sequence identity table of Ty1' Gag,

Ty1c Gag, and Drt2 from strain 227.2. CA-CTD region indicated with white bar below sequence alignment.

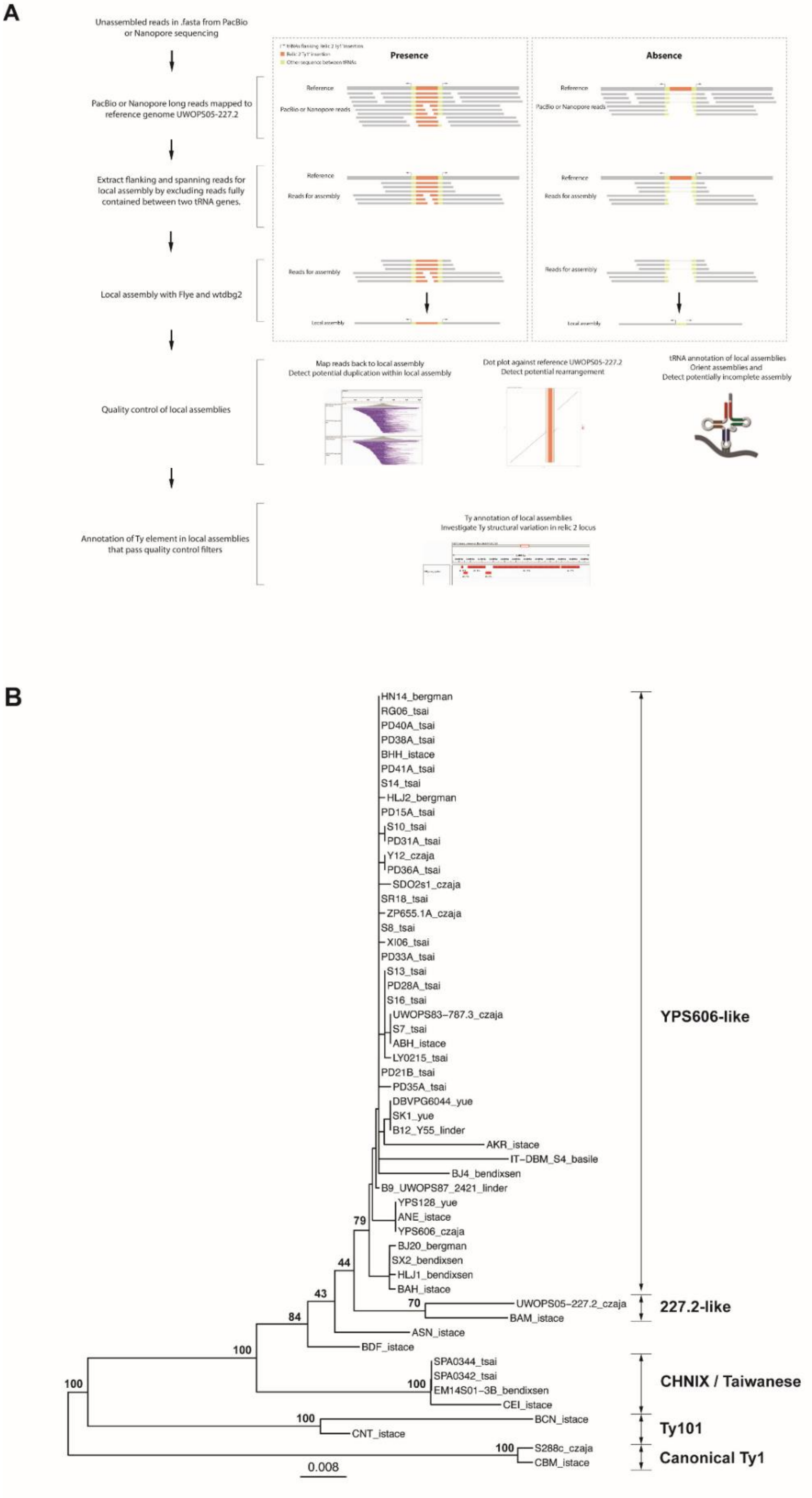

Supplementary Figure S2. Relic 2 locus assembly and quality control workflow and relic 2 phylogeny. A Bioinformatic workflow for assembling the relic 2 locus and checking quality of local assemblies. **B** Neighbor-joining tree of truncated Ty1 family elements in the relic 2 locus.

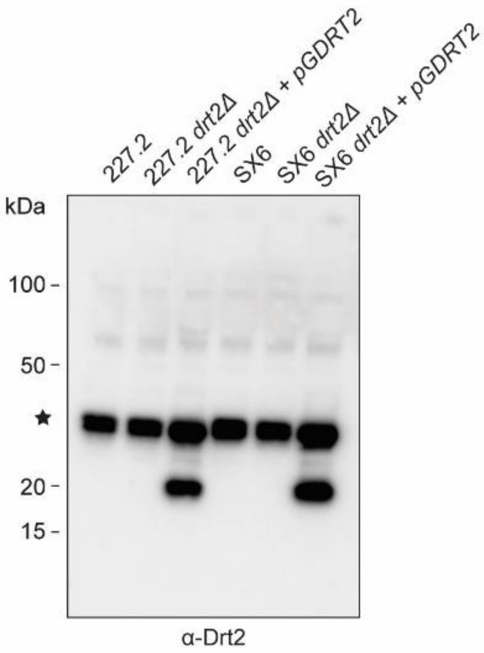

Supplementary Figure S3. Endogenous Drt2 protein levels. Western blot analysis of whole cell extracts with a α-Drt2 primary antibody was used to detect endogenous Drt2 or galactose-induced Drt2 in strains 227.2 and SX6. The black star indicates a non-specific band recognized by the α-Drt2 primary antibody.

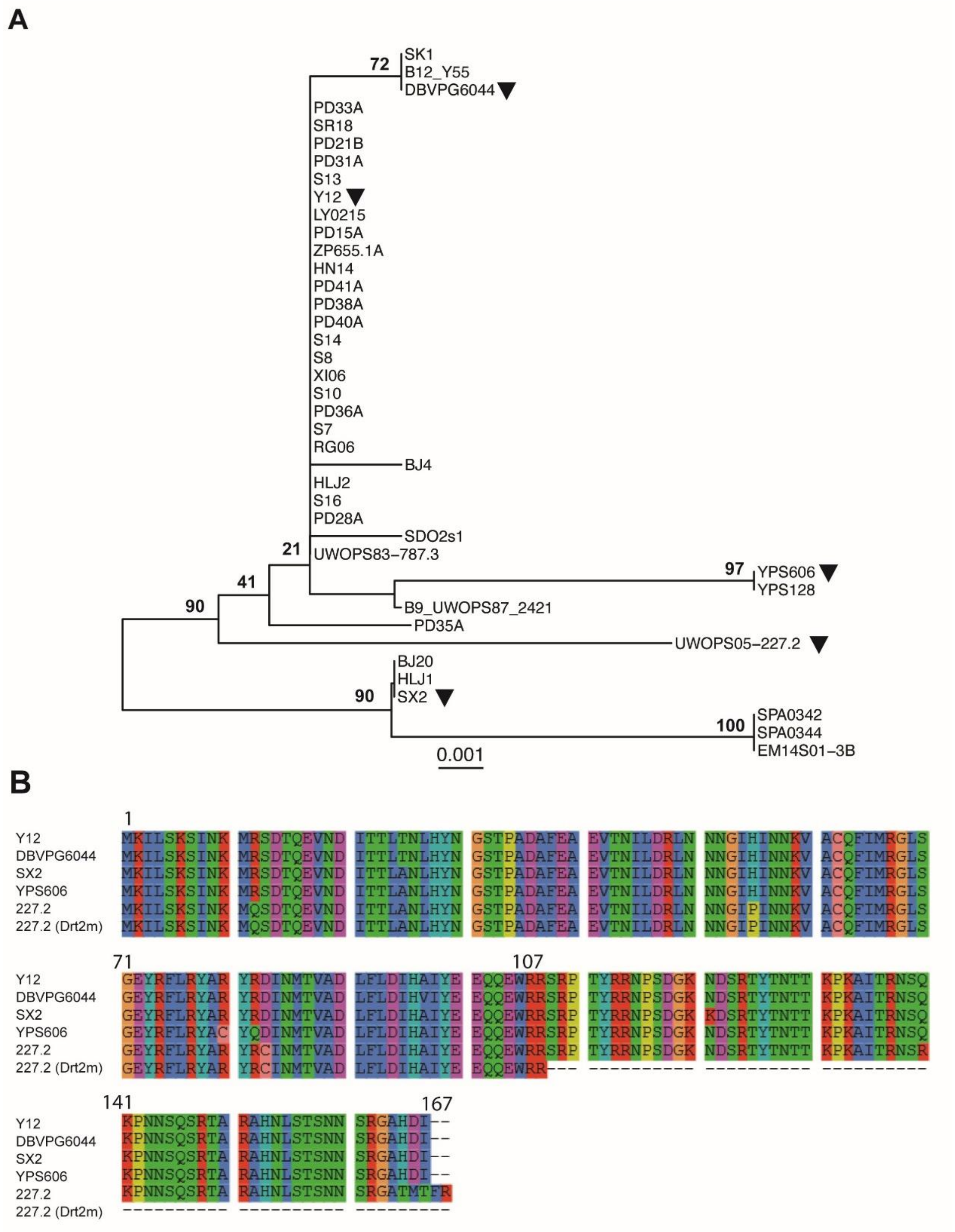

Supplementary Figure S4. *DRT2* CDS phylogeny and *DRT2* construct MSA. A Neighbor-joining tree of the *DRT2* CDS. Strains from which *DRT2* variants were used in Fig. 5 are indicated with a black triangle. **B** Multiple sequence alignment of constructs used in Fig. 5 with length in # of amino acid resides indicated.

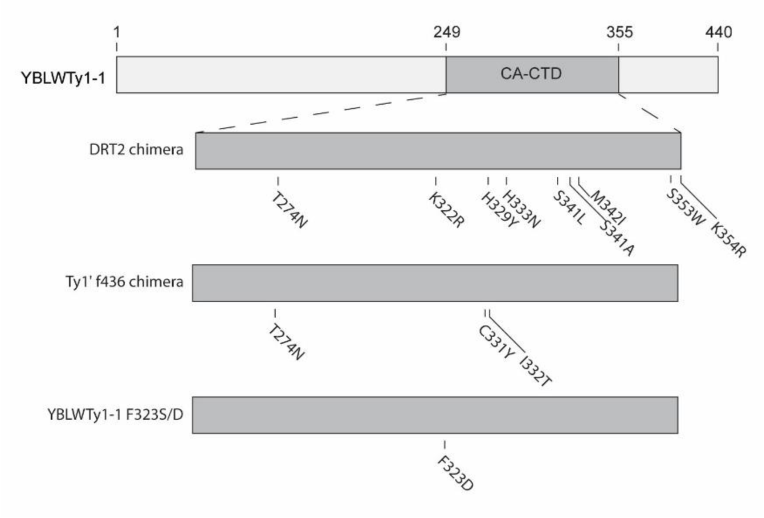

Supplementary Figure S5. Description of chimeric Ty1' reporter elements. Side chain substitutions of chimeric constructs used in Figure 7.

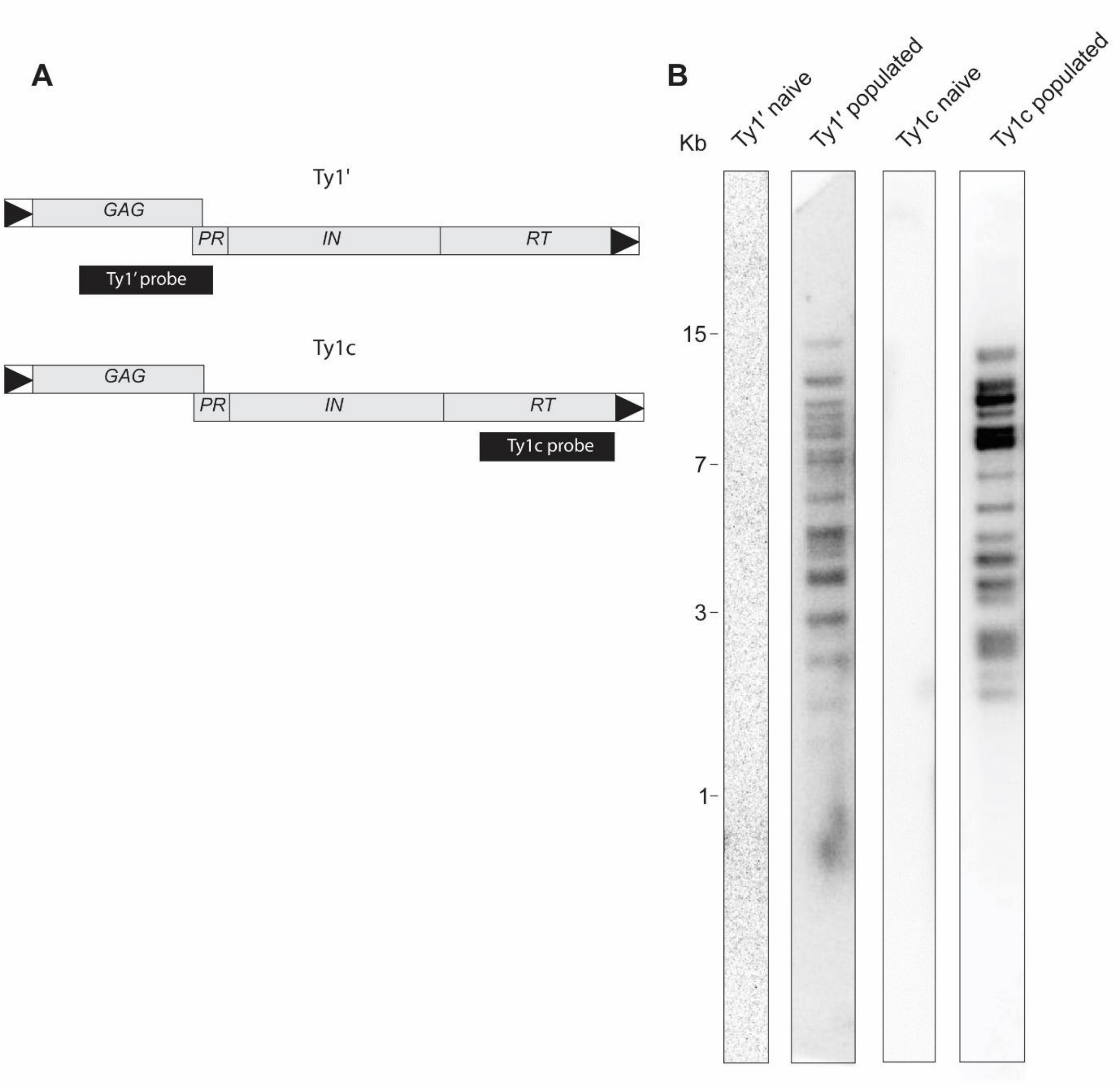

Supplementary Figure S6. Ty1' and Ty1c populated copy number estimate. A Schematic of Ty1' and Ty1c genome with region of probes used for Southern blot analysis. Black triangles indicate long terminal repeats. **B** Southern blot analysis of DNA isolated from populated strains (Fig. 8). For Ty1' copy number estimate the genomic DNA was digested with BglII and separated by 0.6% agarose gel electrophoresis, the blot was hybridized with a ^{32}P-probe generated from the BglII-BbvCI fragment of pGTy1'*his3AI* (pBDG1697). The number of Ty1'-chromosomal junction fragments was used to estimate the number of insertions. The Ty1c copy number estimate was estimated as described previously (Garfinkel, et al. 2003).
